# Construction of Sabatier Volcanoes for CO_2_ Hydrogenation to C1‐2 Oxygenates Using Data‐Efficient Machine Learning

**DOI:** 10.1002/advs.75932

**Published:** 2026-06-16

**Authors:** Mikhail V. Polynski, Sergey M. Kozlov

**Affiliations:** ^1^ Department of Chemical and Biomolecular Engineering, 4 Engineering Drive 4 National University of Singapore Singapore Singapore

**Keywords:** BEP relationships, catalyst design, CO_2_ hydrogenation, heterogeneous catalysis, machine learning, reaction network

## Abstract

The development of technologies for CO_2_ hydrogenation to valuable chemicals is prioritized globally due to their potential for large‐scale CO_2_ abatement. However, rational design of catalysts for this reaction is hindered by an incomplete understanding of the complex reaction network of CO_2_ hydrogenation. This study addresses this gap by establishing a data‐ and computation‐efficient framework for CO_2_ hydrogenation to C1‐2 oxygenates on ≈ 1.2 nm large Au, fcc‐Co, Cu, Ni, Pd, Pt, and Rh nanoparticles. The obtained reaction networks could not be described using Brønsted‐Evans‐Polanyi relationships with sufficient accuracy, motivating the use of a nonlinear NN model for inference of activation energies. A pathfinder‐like algorithm and an energetic span model are used to analyse these networks and to construct Sabatier volcano plots identifying optimal binding properties for a promising catalyst. The analysis indicates that efficient C2 oxygenate formation simultaneously requires CH_x_ formation, favorable C–C coupling and efficient protonation, as well as suppression of methanation and poisoning by CO, alcoholates, and carboxylates. These features are mutually exclusive on the studied monometallic nanoparticles, suggesting that multicomponent and multimetallic catalysts are more realistic design targets. This framework supports computational discovery of CO_2_ hydrogenation catalysts challenged by data limitations and mechanistic complexity.

## Graphical abstract

A new data‐efficient framework combining DFT calculations, a neural network model, and automated graph analysis of catalytic reaction networks is proposed and applied to CO_2_ hydrogenation on transition metal nanoparticles. The analysis shows how efficient C2 oxygenate production requires a balance between CH_x_ formation, C–C coupling, protonation, and poisoning resistance, identifying multicomponent catalysts as realistic design targets.

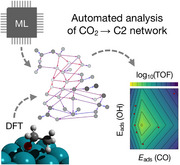

## 1 Introduction

Computational exploration of catalytic reaction networks has achieved unprecedented progress, enabling digital twins for catalytic processes critical for industrial synthesis through detailed insights into reaction mechanisms [1, 2, 3]. Typically, these studies are performed using density functional (DFT) simulations, which offer high accuracy for modeling of main‐group and transition metal systems after careful benchmarking [4, 5, 6]. In turn, microkinetic modeling based on the results of DFT simulations is able to calculate reaction rates within networks with tens to hundreds of elementary steps [1, 7, 8]. The obtained DFT and microkinetic results can be combined with regression machine learning (ML) models to facilitate virtual screening of catalysts [9, 10], or with generative models to optimize catalyst design by predicting promising material compositions [11, 12]. However, this approach is challenged by limited training data for ML models used for predicting catalytic activity, simplistic atomic models misrepresenting realistic active sites in DFT simulations, and the daunting complexity of the comprehensive exploration of the underlying reaction networks. These challenges hinder the predictive accuracy of computational catalyst design studies and their applications to reactions with complex mechanisms [13, 14, 15, 16, 17].

There is an urgent need to address these challenges in the computational catalyst design to accelerate the development of advanced catalysts for CO_2_ hydrogenation toward valuable chemicals. This endeavor is vital for the society, as economically viable CO_2_ utilization could transform the major greenhouse gas into sustainable fuels and materials [18, 19], reconciling environmental protection with continued industrial and technological advancement [20, 21].

Previous computational studies have markedly advanced the development of catalysts for CO_2_ hydrogenation into C_2+_ products, leveraging DFT and machine learning techniques to enhance selectivity and activity. For example, DFT modeling and microkinetic simulations have elucidated mechanisms of complex multistep hydrogenation reactions yielding C_2+_ products, such as the conversion of syngas to linear α‐olefins [22] on Fe‐based catalysts and CO_2_ hydrogenation on Fe‐Co bimetallic catalysts, where controlled C_2+_ yields can be achieved via graphene fencing [23]. Machine learning can be used to explain what reaction conditions and catalyst features (e.g., catalyst support, metal, and promoter) drive C_2+_ hydrocarbon selectivity [24], narrowing the experimental search for catalysts with desired properties. Computational modeling has also rationalized the activity of novel catalysts for CO_2_ hydrogenation, such as CeO_2_‐supported diatomic palladium sites, with nearly 100% ethanol selectivity [25]. However, previous studies provided limited training data for machine learning models facilitating the exploration of the CO_2_ hydrogenation mechanism in sufficient detail [26]. Indeed, only a handful of previous studies explored the reaction network of CO_2_ hydrogenation to multicarbon products systematically and comprehensively enough to generate a sufficient data set for the training of predictive ML models for this reaction [27]. However, such studies are typically limited only to a single transition metal, e.g., Cu, and are hard to extrapolate to other materials for the purpose of rational catalyst design.

In this study, we propose several methodological innovations to address these gaps and apply them to the construction and analysis of the reaction network for CO_2_ hydrogenation to C1 and C2 oxygenates. First, we developed a data‐efficient regression neural network architecture rooted in revised Brønsted‐Evans‐Polanyi relationships with explicit account for the reaction mechanism in each elementary step. The proposed methodology also explicitly accounts for co‐adsorption energies of reacting species. Additionally, we evaluate the benefits and challenges of applying a fast shortest‐path‐based algorithm for automatic analysis of reaction graphs, the energetic span model [28], and microkinetic modeling to mechanistically complex processes such as CO_2_ hydrogenation, creating a platform for the rational catalyst design workflow. Using the proposed approach, we constructed Sabatier volcano relationships for CO_2_ hydrogenation into methane, ethanol, and acetic acid. These volcanoes allowed us to identify the binding properties of the Sabatier‐optimal catalyst that would result in the highest catalyst activity in these reactions and evaluate how far the activities of the considered transition metals are located from the Sabatier maximum.

## 2 Results and Discussion

### 2.1 Catalytic Reaction Networks

**FIGURE 1:**
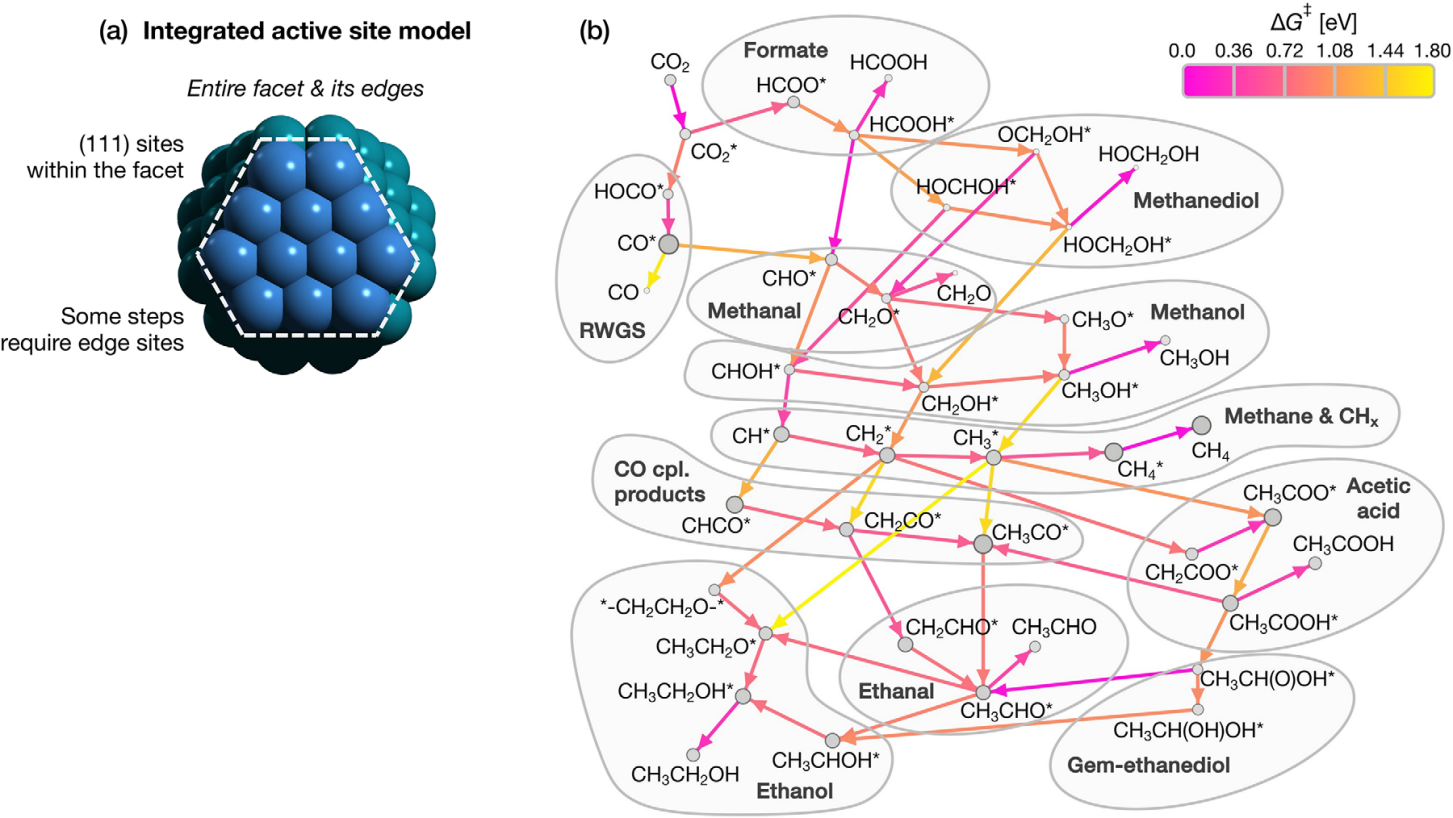
Modeling approach: (a) integrated active site model; (b) adopted reaction network of CO_2_ hydrogenation. The size of the node circles reflects the relative stability of various intermediates on Rh NPs, measured by Δ*G_n_*, where the largest nodes indicate the highest stability and vice versa. Edge colors indicate the free activation energies (Δ*G*‡) of elementary steps on Rh NPs, chosen as a representative example.

We adopted the catalytic reaction network for CO_2_ hydrogenation into C1 and C2 oxygenates previously explored for the case of Cu and Pd [29] and expanded the considered catalyst set by modeling cuboctahedral fcc M_79_ nanoparticles of Au, Co, Ni, Pt, and Rh. The considered reaction networks included intermediates and products such as CO, formic acid, methanediol (unstable, but relevant in some hydrogenation pathways), methanal, methanol, and unwanted methane, as well as key C2 products such as acetic acid, ethanal, and ethanol. Kinetic and thermodynamic parameters were obtained using a combination of DFT calculations and predictions from a trained ML model (see computational details in Notes S1–S3). An integrated active site representation (Figure 1a) was used to capture the diversity of possible active centers on nanoparticle surfaces, encompassing typical discrete adsorption sites such as top, bridge, and hollow sites, as well as nanoparticle edges and near‐edge regions. Note that we explicitly include co‐adsorption energies and additional factors, such as the post‐reaction orientation of the product, in our modeling framework to enable thermodynamically consistent integration of elementary steps into the reaction network [29]. Complete numerical data are provided in the Supporting Spreadsheet. Here, we illustrate the calculated network of CO_2_ hydrogenation to C2 products using the Rh nanoparticle as an example (Figure 1b).

Figure 1b displays the reaction network as a directed graph, with larger nodes indicating more negative relative free energies (Δ*G_n_*) of intermediates under standard conditions, referenced to isolated gas‐phase CO_2_ and H_2_. In turn, edge colors indicate the free activation energies (Δ*G* ^‡^) of elementary steps calculated as the differences between the free energies of transition states and the respective initial states. Given the complexity of the reaction (encompassing over 40 elementary steps examined across seven metallic surfaces), we use automated analysis of the reaction networks on these surfaces (see details in Notes S4–S6). Below, we adopt a higher‐level perspective, systematically analyzing the catalytic activities of the metals within each type of elementary step. In turn, representative reaction pathways are discussed in Note S7.

### 2.2 BEP Relationships

The catalytic network encompasses four elementary step categories, including C–O cleavage and C–C coupling reactions, which are essential for the reduction of carbon centers and the formation of multicarbon products. Two other categories involve the transfer of H atoms: hydrogenation of carbon atoms (e.g., CH* + H* ⟶ CH_2_*) and protonation of oxygen atoms to form surface‐bound intermediates bearing Brønsted‐acidic functional groups such as ‐OH and ‐COOH, e.g,

$$\mathrm{HCO}O^{*}+H^{*}\to \mathrm{HCOO}H^{*}$$

$$CH_{3}O^{*}+H^{*}\to CH_{3}OH^{*}$$

For these steps, the performed DFT calculations were limited to key transformations, because the total number of hydrogenation and protonation steps in the reaction network (Figure 1b) exceeds the combined number of C–C coupling and C–O cleavage steps by approximately a factor of two (see data in the Supporting Spreadsheet). Moreover, these steps can be reliably described using the Brønsted‐Evans‐Polanyi (BEP) relationships outlined in our previous study [29].

**FIGURE 2:**
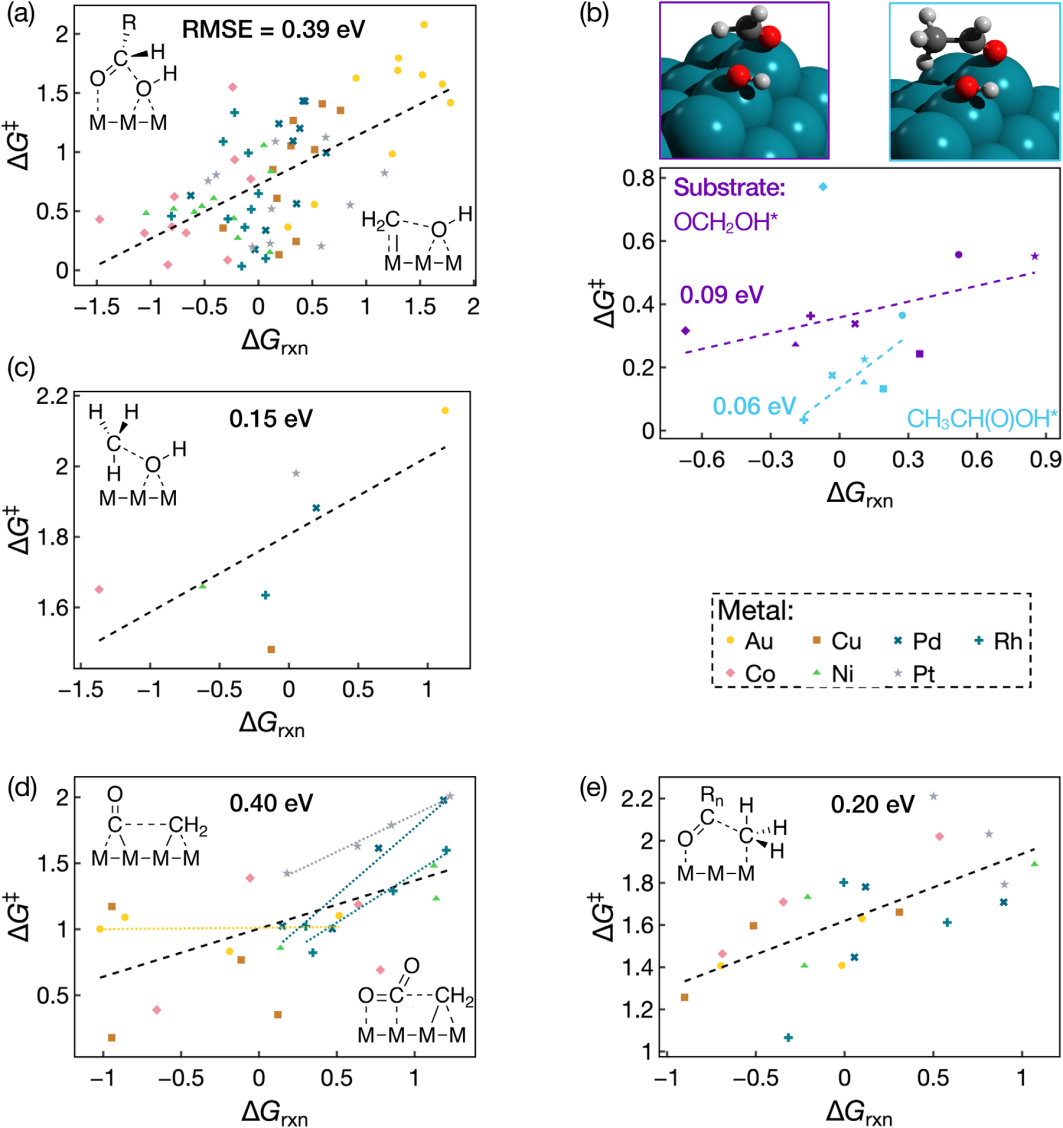
Brønsted–Evans–Polanyi relationships for key elementary steps in the CO_2_ hydrogenation network (RMSE values are shown for each fit of all data points): (a) C–O cleavage steps representing reversed OH nucleophilic addition to C(sp^2^) for all species; (b) BEP relationships for C–O cleavage only in OCH_2_OH* and CH_3_CH(O)OH*; structures of the TS on Rh are also shown (TS on other metals are structurally identical); (c) homolytic C–O bond cleavage; (d) metathesis‐like C–C coupling involving CH_2_* and CH* species; (e) C–C coupling involving addition of CH_3_* to C(sp^2^). Chemical structures represent common TS structures. Black dashed lines represent linear fits to all data in each panel; colored dotted lines correspond to metal‐specific fits with the same colors as the respective metal markers. Jmol coloring was used in all panels except (b); distinct symbols denote metals, as shown in the legend above (e). All values are in eV. All fitting parameters are listed in Table S1.

Our analysis of transition state structures for C‐O cleavage on Cu, Pd, Au, fcc‐Co, Ni, Pt, and Rh revealed that most of the steps, such as:

(1) $$\mathrm{HCOO}H^{*}\to \mathrm{CH}O^{*}+OH^{*}$$

(2) $$\mathrm{OC}H_{2}OH^{*}\to CH_{2}O^{*}+OH^{*}$$

(3) $$CH_{3}\mathrm{CH}\left(O\right)OH^{*}\to CH_{3}\mathrm{CH}O^{*}+OH^{*}$$

can be viewed as reversed nucleophilic additions of OH* to a double‐bonded carbon (i.e., to C─O; see Figure 2a).

Figure 2a demonstrates a general upward BEP trend where activation and reaction energies increase simultaneously with the metal electronegativity, progressing from more electropositive Co and Ni through Rh, Pt, Cu, and Pd, to the relatively electronegative Au. This aligns with the expected general tendency of electropositive metals to abstract nucleophilic OH^−^ more easily, although the overall RMSE for this linear BEP fit is high (0.39 eV). For example, metals such as Cu and Pd exhibit slopes markedly deviating from the general trend. Additionally, within individual metals (e.g., Co and Pt), data points occasionally follow non‐linear patterns, while for Rh, activation energies appear largely independent of Δ*G_rxn_*.

While most C–O cleavage steps share a similar TS structure and comprise a high‐scatter BEP relationship, Figure 2b illustrates that selecting only one *specific* C–O cleavage step and varying the metal may sometimes yield a clearer linear BEP correlation. In this case, the overall RMSE values of the obtained BEP become notably low, at 0.09 and 0.06 eV for methanal and ethanal formation, respectively, with Cu and Co being the most notable outliers for each reaction, respectively. At the same time, the slopes of linear fits for reactions (2) and (3) differ significantly (0.17 and 0.55, respectively), as do their intercepts (0.36 and 0.14, respectively). This finding is rather surprising because these two reactions differ solely by one substituent (H versus CH_3_) and involve similar TS structures; therefore, they would be expected to follow the same BEP and scaling relationships [30, 31, 32]. The emergence of distinct BEP relationships for homologous reactants will have critical implications for the computational catalyst design process and will be addressed in more detail below.

The remaining C–O cleavage steps are identified as homolytic [29], exhibiting a distinct TS structure depicted schematically in Figure 2c for the step:

(4) $$CH_{3}OH^{*}\to {\mathrm{CH}}_{3}^{*}+OH^{*}$$

Clearly, a BEP‐type correlation for this step emerges when varying the nature of the metal catalyst, with an RMSE as low as 0.15 eV, and Cu being a noticeable outlier from the linear trend (Figure 2c). This linear relationship shows an increase of Δ*G* ^‡^, when going from more electropositive metals (Co, Ni) to more electronegative ones, with Au exhibiting the highest barrier. That is, more electropositive metals are calculated to more readily abstract nucleophilic OH^−^.

The next reaction class to evaluate within the BEP framework comprises the C–C coupling steps. Figure 2d illustrates the distribution of data points for a specific class of C–C couplings, which proceed via a metathesis‐like transition state. In the current analysis, combining data for Au, Cu, fcc‐Co, Ni, Pd, Pt, and Rh does not produce a clear linear correlation, resulting in a high RMSE of 0.40 eV. However, examining heavier metals (Au, Pd, Pt, and Rh) individually yields distinctly linear relationships, as shown in Figure 2d. Additionally, in Note S2, we demonstrate that selecting a particular C–C coupling step (such as the coupling of CH_2_* with CO or CO_2_) and combining data points across all investigated metals may fail to yield a clear linear BEP correlation. In turn, another subclass of C–C coupling steps, representing nucleophilic addition to carbon in C─C or C─O moieties, proceeds via a Felkin‐Anh‐like transition state and displays a linear relationship between Δ*G* ^‡^ and Δ*G_rxn_* with an RMSE of 0.20 eV (Figure 2e).

From the discussion above, we conclude that there appears to be no universal principle governing the precise grouping of elementary steps into a single BEP relationship. Factors such as metal identity (electropositive Ni vs. electronegative Au), reaction class (e.g., C–O cleavage or C–C coupling), transition state structure (e.g., metathesis‐like vs. Felkin‐Anh‐like TS), and the molecular structures of reactants and products may all influence the activation barriers. This observation pushed us to develop a more refined approach for rapid estimation of activation barriers, which is capable of distinguishing metal identity, transition state type, and the structures of reacting species. In particular, H* association steps were exhaustively calculated in our previous work [29] on CO_2_ hydrogenation on Cu and Pd and are not thoroughly modelled in this study; instead, their barriers are inferred using ML models trained on previously obtained data and additional DFT calculations as discussed below.

### 2.3 Data‐Efficient Machine Learning

To train an ML model for the prediction of activation barriers in CO_2_ hydrogenation with consistent accuracy across all reaction types and metals, we assembled a dataset of all DFT‐calculated Δ*G_rxn_* and Δ*G* ^‡^ for C–C coupling, C–O cleavage, hydrogenation, and protonation steps on Au, Cu, fcc‐Co, Ni, Pd, Pt, and Rh. This dataset underwent a stratified split into training and test sets to obtain a representative test set capable of reliably estimating model performance while retaining sufficient diversity. Comprehensive details on training procedures and model performance are provided in Note S3, while the dataset and predicted values are listed in the Supporting Spreadsheet. Here, we highlight only key findings and methodological aspects.

**FIGURE 3:**
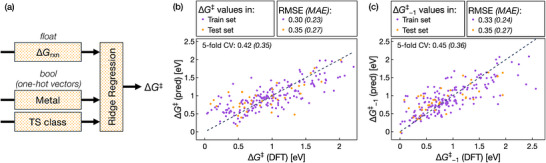
“Generally linear” BEP‐like model: (a) model features; (b) parity plot for Δ*G* ^‡^ values in forward steps; (c) parity plot for Δ*G* ^‡^ values in reverse steps (denoted as $\Delta G_{-1}^{‡}$ for clarity). Dashed lines in (b) and (c) represent ideal agreement between DFT‐calculated and ML‐predicted values; MAE values in (b) and (c) values are given in italic font.

Initially, we assessed the viability of a “generally linear” ridge regression model, incorporating Δ*G_rxn_*, metal identity, and transition state class as input features. Here and in the other trained models, metal identity and transition‐state classes were encoded using one‐hot vectors (Figure 3a). Parity plots illustrating model predictions for forward and reverse activation barriers across elementary steps are presented in Figure 3b, c, alongside model accuracy metrics calculated for training, test, and 5‐fold cross‐validation runs.

The linear ML model exhibited suboptimal accuracy on the test set, achieving RMSE values of ≈ 0.30 eV, which is close to the RMSE previously reported for BEP relationships lacking detailed structural encoding and BEP‐like linear models [33]. Moreover, the model showed poor performance in cross‐validation (with RMSE = 0.42 eV and MAE = 0.35 eV, 5 folds), essentially exhibiting the same accuracy as the conventional linear BEP fits presented earlier (Figure 2a, d). Notably, transitioning to a nonlinear gradient boosting approach, such as the CatBoost algorithm [34] (discussed further in Note S3), with identical input features provided only marginal gains in accuracy. These findings support our hypothesis that highly accurate ML models for rapid prediction of reaction barriers should explicitly incorporate the reactant and product structures.

**FIGURE 4:**
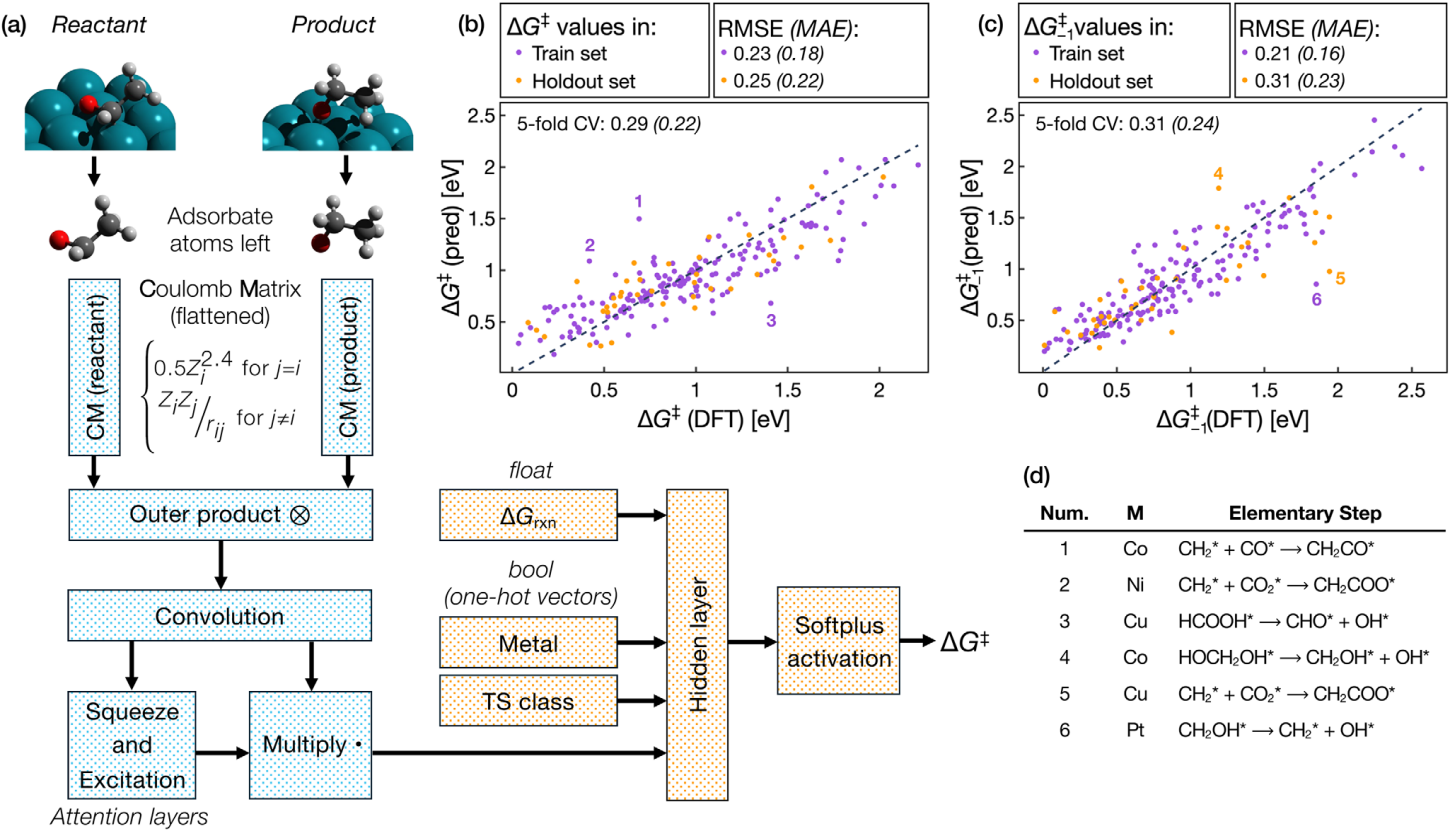
Neural network model for inference of activation barriers: (a) simplified representation of the model architecture; (b) parity plot for Δ*G* ^‡^ values in forward steps for the best‐performing model; (c) parity plot for Δ*G* ^‡^ values in reverse steps for the best‐performing model; (d) details on outlier steps highlighted by numbers in panels (b) and (c). Dashed lines in (b) and (c) represent ideal agreement between DFT‐calculated and ML‐predicted values; MAE values in (b) and (c) are given in italic font. For the detailed representation of the model architecture and statistics over 20 training runs, see Note S3. *Z_i_* and *r_ij_* denote nuclear charges and interatomic distances, respectively.

The structural details of reactants and products were explicitly accounted for by employing a neural network (NN) designed for the scarce training data regime. As illustrated in Figure 4a, the NN architecture comprises distinct input branches. Coulomb matrices representing isolated reactant and product structures are combined using an outer product, yielding a 100 × 100 × 1 interaction map. A two‐dimensional convolutional (Conv2D) layer processes this interaction map, followed by a block featuring the squeeze‐and‐excitation attention mechanism [35]. Subsequent pooling produces a 10‐component vector. The other branches include metal identity and TS class, each encoded using one‐hot encoding (as previously used in the linear model), and processed by dense layers. Reaction free energy, Δ*G_rxn_*, is directly incorporated into the NN model as a scalar. Concatenation of these feature sets is fed into a 10‐neuron dense layer, followed by a single‐neuron output layer utilizing the softplus activation function to ensure positive predictions of Δ*G* ^‡^, which is a valuable feature not straightforwardly achievable with linear or gradient boosting models. Given inherent stochasticity due to random weight initialization leading to models of differing accuracy, we performed 20 independent training runs and report mean values and standard deviations of RMSE and MAE across train and holdout datasets, along with the metrics obtained during cross‐validation (Note S3). A detailed description and justification of the chosen NN architecture are provided in Note S3.

Figure 4b demonstrates accuracy and generalizability improvements for predicting forward Δ*G* ^‡^, achieving mean RMSE values on the holdout set (0.25 eV for the final NN model selected for inference), which are notably better than those obtained by linear models (0.35 eV). A distinctive general strength of the obtained NN models is the minimal discrepancy between MAE on holdout and cross‐validation sets, which indicates robust generalization and accurate capture of structural features despite the limited training dataset. Although the RMSE on the training set (0.23 eV) is modestly lower than that on the holdout (0.25 eV), the holdout was drawn by stratified sampling over every reaction class and metal combination to ensure that even the less common hydrogenation and protonation steps appear in the testing set. At the same time, the obtained NN models achieve similar RMSE and MAE values of 0.21 and 0.19 eV, respectively, for predicting hydrogenation barriers within the holdout subset for underrepresented Au, fcc‐Co, Ni, Pt, and Rh without significant outliers (see Supporting Spreadsheet). All major prediction errors in Figure 4b predominantly belong to C–C coupling or C–O cleavage steps, which are inherently challenging to correlate with any descriptors (Figure 4d). Therefore, the obtained NN model significantly enhances prediction accuracy and generalizability, compared with the linear BEP‐like model analyzed in Figure 3a.

Similarly, Figure 4c highlights a performance improvement for reverse Δ*G* ^‡^ prediction. While there is an increase in its RMSE on the holdout set (0.31 eV) due to two notable outliers, compared to the RMSE of the NN model for forward barriers (0.25 eV), there is still a significant improvement in accuracy when using the optimal NN model, compared to the simple linear counterpart (0.35 eV, Figure 3). Clear outliers in the parity plot for reverse barriers (Figure 4d) again correspond exclusively to reaction classes that are challenging to predict (C–C coupling and C–O cleavage steps). Importantly, there is no degradation in relative predictive accuracy when moving from forward to reverse barrier prediction, as indicated by the identical ratio of the Δ*G* ^‡^ range to the RMSE of the NN models for forward and reverse steps (1.17 in both cases). Moreover, accuracy for underrepresented hydrogenation reactions on Au, fcc‐Co, Ni, Pt, and Rh is encouragingly high, with RMSE and MAE values of only 0.18 and 0.15 eV, respectively (see the Supporting Spreadsheet for values).

In summary, the implemented NN models exhibit significantly higher accuracy of reaction barrier predictions, effectively overcoming the limitations inherent to the simplistic linear BEP‐like models by explicitly encoding the molecular structures of reactants and products, as well as the transition state classes. The NN delivers notably superior prediction accuracy, even for hydrogenation and protonation steps underrepresented in the training data. The proposed model, thus, addresses critical issues such as small datasets, structurally diverse reaction classes, and metal‐dependent variations in activation energies. Given these advantages, the trained NN models were employed to infer activation barriers for hydrogenation and protonation elementary steps within the CO_2_ hydrogenation reaction network. These steps collectively outnumber C–C coupling and C–O cleavage steps by approximately a factor of two. Thereby, the NN models substantially reduced the computational cost of this study relative to exhaustive DFT calculations.

### 2.4 Estimating Catalytic Activity of Metal Nanoparticles

Establishing the comprehensive catalytic reaction network as well as ML models for rapid prediction of reaction barriers allowed us to systematically evaluate catalytic activity trends for the production of the main high‐value C2 oxygenates, ethanol and acetic acid, through CO_2_ hydrogenation (see details in Note S7). Additionally, we evaluated the activity of the considered metals, Au, fcc‐Co, Cu, Ni, Pd, Pt, and Rh, in the CO_2_ hydrogenation to methane, which is considered a low‐value byproduct, detrimental to the techno‐economic performance of many catalytic processes. C1 products (CO, HCOOH, CH_2_O, CH_3_OH) and ethanal are discussed in Note S5. Here, we assume a steady‐state catalytic regime, where key intermediates can populate the surface sufficiently to sustain all relevant reaction steps without poisoning the catalyst surface. In turn, potential poisoning effects are evaluated from the perspective of excessively exothermic formation energies Δ*G_n_* for certain intermediates in the reaction network, which increase the energetic span of pertinent CO_2_ hydrogenation pathways.

To analyze the reaction network, we implemented a revised version of the Pathfinder algorithm [36], which allowed us to identify kinetically favorable reaction pathways for each target product under the chosen reaction conditions. The mechanisms obtained in this way were then analyzed within the energetic span model [28] to estimate TOF values and identify the TOF‐determining intermediate (TDI) and transition state (TDTS), using the degree of TOF control. This approach provides a physically interpretable and computationally efficient way to rank candidate mechanisms, incorporating both kinetic barriers and reagent availability, and mapping a large reaction network onto mechanistically clear cycles with interpretable TOFs. Finally, the calculated TOFs were used to construct the Sabatier volcanoes for CO_2_ hydrogenation to selected products and to compare catalytic activities across metals. A comprehensive discussion of the algorithm implementation and application is provided in Notes S4–S7.

For activity estimations, we simulated the supply of feed gases with p(H_2_) = 30 bar and p(CO_2_) = 10 bar at T = 523.15 K, which is a common condition for the synthesis of C_2+_ oxygenates via CO_2_ hydrogenation [37]. In addition, we considered an alternative scenario in which all relevant gas‐phase species (CO, HCOOH, CH_2_O, CH_3_OH, CH_4_, CH_3_COOH, CH_3_CHO, and CH_3_CH_2_OH) are present in the gas phase at a pressure equal to 1 bar to emulate operation under conditions, when substantial amounts of products have already accumulated in the gas phase; this scenario yields slightly different optimal mechanisms, which are analyzed in detail in Note S7. The associated mechanistic discussions, along with tabulated TDI and TDTS, for all metals are also given in Note S7.

**FIGURE 5:**
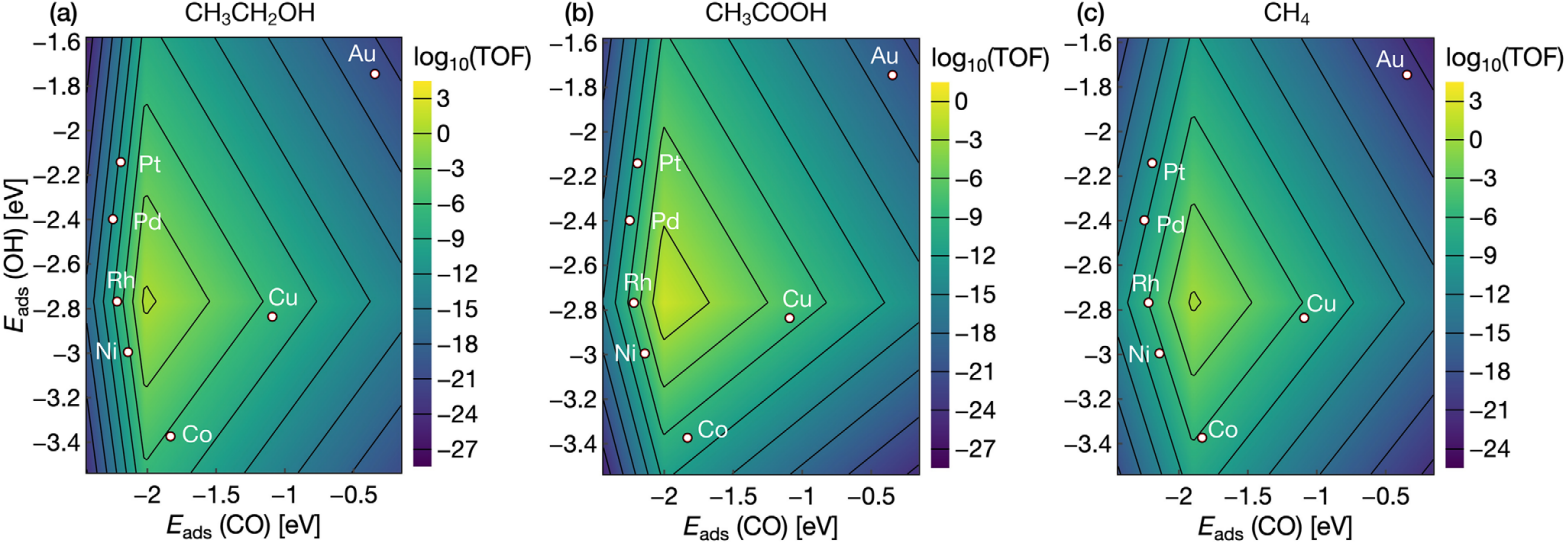
The obtained two‐dimensional Sabatier volcano plots at p(H_2_) = 30 bar and p(CO_2_) = 10 bar at T = 523.15 K for catalyst activity in CO_2_ hydrogenation to (a) ethanol, (b) acetic acid, and (c) methane as a function of binding energies towards OH and CO species. Contour plots are fitted surfaces. All computed log_10_(TOF) values are given in Table S11. Δ*E_ads_* (CO) and Δ*E_ads_* (OH) are CO and OH adsorption energies, respectively; more negative values correspond to stronger binding.

Previous studies demonstrated that the activity of transition metal catalysts in reactions involving CO or CO_2_ typically correlates with the binding energies of two species [38, 39, 40, 41], which are usually chosen to represent carbon‐bound fragments (e.g., CO* or CH_x_*) and oxygen‐bound fragments (e.g., OH*). Here, we additionally considered dissociative adsorption energy of hydrogen as a potential descriptor of the overall activity. Among all considered combinations, CO and OH adsorption energies, Δ*E_ads_* (CO) and Δ*E_ads_* (OH), delivered the most accurate Sabatier volcano fits and physically meaningful fitting parameters (note that more negative values correspond to stronger binding). To correlate predicted TOF with two binding energies Δ*E_ads_* (*CO*) (taken as *x*) and Δ*E_ads_* (*OH*) (taken as *y*), we employed the pyramid‐like volcano relationship:

$$\begin{array}{ccc} \mathrm{lg}\left[TOF\left(x,y\right)\right] & = & \mathrm{lg}\left(TOF^{opt}\right)-\max\left[a_{x}^{+}\left(x-x_{0}\right),a_{x}^{-}\left(x_{0}-x\right)\right]\\ & & -\max\left[a_{y}^{+}\left(y-y_{0}\right),a_{y}^{-}\left(y_{0}-y\right)\right], \end{array}$$

where *x*_0_ and *y*_0_ define the location of the Sabatier optimum (the apex of the volcano). In turn, $a_{x}^{+}$, $a_{x}^{-}$, $a_{y}^{+}$, and $a_{y}^{-}$ represent slopes for various sides of the volcano, which often differ [38, 42]. For example, the slopes on strongly binding sides of the volcano may be strongly affected by catalyst poisoning by some intermediates. Finally, *TOF^opt^* approximates the highest achievable TOF on the considered Sabatier volcano. In addition, this approach leverages the inherent linearity of the underlying BEP and free energy scaling relationships. Figure 5 presents calculated TOF values and two‐dimensional Sabatier volcano plots for key C2 oxygenates, ethanol, and acetic acid (ethanal is discussed in Note S6), and the undesired methane byproduct as a function of CO and OH binding energies. Note that the same descriptors were also used in a previous study targeting CO hydrogenation into similar products [40].

Table 1 summarizes the general catalytic activity trends derived from reaction networks modeled via the combined DFT‐ML methodology described in previous sections. A notable similarity in the volcano plots (Figure 5a, b) for the main C2 products and CH_4_ (Figure 5c) arises primarily because the majority of TOF‐determining steps in all three pathways involve either the formation of CH_x_ surface species (necessary in the production of both C2 products and CH_4_) or protonation of carboxylates, HCOO* or CH_3_COO*. The former surface carboxylate species was the intermediate involved in the majority of predicted mechanisms, while the latter was the necessary intermediate in the formation of C2 products on some of the considered metals. Such common features in the reaction mechanisms for CO_2_ hydrogenation to various products result in small differences between Sabatier‐optimal Δ*E_ads_* (CO) and Δ*E_ads_* (OH) values on all calculated volcanoes. In particular, Table 1 highlights that the apexes of the Sabatier volcanoes for CO_2_ hydrogenation to ethanol and acetic acid (as well as CH_3_CHO, Table S6) are very close to each other, indicating that these C2 oxygenates share a common Sabatier‐optimal catalyst. In contrast, the Sabatier‐optimal catalyst for methane formation is distinct: while the optimal Δ*E_ads_* (OH) binding energy remains similar, the optimal Δ*E_ads_* (CO) is located in the region of weaker binding. Note that estimated TOF values at the apexes of all three constructed volcanoes vary between tenths of s^−1^ and several s^−1^, which is in line with 10^−3^ to 1 s^−1^ TOFs typically observed in CO_2_ hydrogenation [43, 44, 45, 46, 47]. While differences between the peaks in the obtained Sabatier volcanoes may appear insignificant, they have strong implications for catalyst selectivity. Indeed, a catalyst with optimal Δ*E_ads_* (CO) and Δ*E_ads_* (OH) for ethanol synthesis would exhibit 336: 9: 1 selectivity for ethanol, acetic acid, and methane, respectively, according to the TOFs estimated from the obtained volcano relationships. Note that the RMSE of the pyramidal fits introduces some uncertainty in the predicted TOF values (Table 1), which is unavoidable, however, in computational catalyst screening due to the finite accuracy of the underlying computational approaches.

**TABLE 1:** Sabatier‐optimal catalyst descriptors and performance metrics for p(H_2_) = 30 bar and p(CO_2_) = 10 bar at T = 523.15 K.

Product	CH_3_CH_2_OH	CH_3_COOH	CH_4_
Δ*E_ads_* (CO), eV	−2.02	−2.01	−1.90
Δ*E_ads_* (OH), eV	−2.77	−2.77	−2.77
*TOF^opt^*, s^−1^	4.28	0.24	3.08
RMSE $\log_{10}\left(TOF^{opt}\right)$	1.50	1.46	2.48

Gold, as the most weakly binding catalyst, exhibits very low TOF ≈ 10^−20^ s^−1^ for the C2 products and CH_4_ at the specified conditions (see Table S7 for all numerical values). In turn, the somewhat more strongly binding nature of Cu particles results in much higher TOFs around 10^−8^ s^−1^, although they still exhibit an empty surface as the resting state (Table S11). The nanoparticles of fcc‐Co exhibit even higher TOF of 10^−6^ s^−1^ despite their highly oxophilic properties, which result in overstabilization of carboxyl intermediates. In turn, the activity of Pd and Pt catalysts is undermined by their exceedingly strong binding of CO* molecules, resulting in CO poisoning under reaction conditions. Among moderately binding catalysts, such as Rh and Ni, the former is the closest to the apex of the volcanoes for CO_2_ hydrogenation into ethanol, acetic acid, and methane (Table 1).

Although Rh is the closest transition metal to the apex of the volcanoes for ethanol and acetic acid production, its estimated TOFs are as low as 1.03 · 10^−5^ and 1.0 · 10^−6^ s^−1^, respectively. This observation aligns well with experimental evidence, where efficient CO_2_ hydrogenation to C2 products predominantly requires multimetallic catalysts [37], although some evidence indicates that promoted Rh catalysts may also facilitate C2 oxygenate production [48, 49]. At the same time, our analysis predicts that Rh nanoparticles are much more active in CO_2_ methanation, with an estimated TOF of 1.24 · 10^−2^ s^−1^ for CH_4_ formation. This behavior is fully consistent with kinetic studies on conventional Rh/Al_2_O_3_ catalysts, which display quantitative selectivity to methane and turnover frequencies for CH_4_ formation on the order of 10^−2^ s^−1^ at 200°C [44]. Therefore, our analysis reproduces the tendency of unpromoted Rh nanoparticles to favor methanation over the synthesis of C2 oxygenates. Moreover, our analysis also suggests why promoters, tailored supports, or tandem architectures are required to redirect Rh‐based catalysts toward more valuable C2 products.

At this point, it is important to critically compare the proposed approach to more established microkinetic simulations of surface reactions, which are often used for the quantification of activities and selectivities of applied catalysts [16, 50]. One of the main advantages of the developed combination of the Pathfinder‐like algorithm and the energetic span model is its ability to evaluate the catalyst activity towards the formation of any product — provided that the assumptions underlying the model hold (namely, the dominance of a single formation mechanism for a specific product and the exergonicity of product formation). For example, our microkinetic simulations show that the rate of ethanol production on Rh catalysts is 1.46 · 10^−18^ s^−1^, because they hydrogenate CO_2_ exclusively into minor quantities of HCOOH (rate equal to 1.42 · 10^−4^ s^−1^), which rapidly desorbs from the simulated active sites instead of further hydrogenation. Similarly, microkinetic simulations would predict near‐zero rates for CO_2_ hydrogenation to C2 products on most of the catalysts considered, thereby preventing the construction of a meaningful Sabatier volcano plot for these reactions. In practice, CO_2_ hydrogenation may involve readsorption and further reduction of gas‐phase intermediates such as HCOOH, CO, CH_2_O, and others as discussed in our previous study [51]. Therefore, to simulate such processes consistently, multiscale modeling approaches rather than conventional microkinetic modeling are required.

In view of such complex multi‐scale nature of CO_2_ hydrogenation processes, the obtained product‐specific Sabatier volcanoes in Figure 5 should therefore be interpreted as screening‐level activity maps. In this context, the Pathfinder‐like selection of kinetically favorable pathways and their energetic span analysis were used to identify the descriptor regions where each target product could be formed efficiently, without requiring an often challenging comprehensive multiscale kinetic simulations. In turn, microkinetic simulations can be used at a later stage to gain even more information on the best‐performing catalyst and, if necessary, refine the model used for catalyst screening.

For the sake of comparison, we performed microkinetic simulations of CO_2_ hydrogenation into C2 products on Rh by removing the desorption steps for certain intermediates from the system of microkinetic equations (see details in Note S8). In essence, disallowing the desorption of certain species is equivalent to assuming that the rate of their desorption equals the rate of their readsorption from the gas phase, which contains a quasi‐equilibrated concentration of these compounds. Our microkinetic simulations conducted in this way at T = 523.15 K yielded the production rate of ethanol on Rh to be 3.35 · 10^−5^ s^−1^, just slightly higher than TOF = 1.03 · 10^−5^ s^−1^ obtained using the energetic span model on the automatically found most kinetically probable mechanism. For the undesired product, CH_4_, our model predicts a TOF of 1.24 · 10^−2^ s^−1^, while on Al_2_O_3_‐supported Rh nanoparticles, TOFs for CO_2_ hydrogenation to CH_4_ have been reported to be on the order of 10^−2^ at 200°C [44],

The obtained Sabatier‐optimal TOF value for ethanol production (4.28 s^−1^ in Table 1) is close to the previously reported experimental TOF of 0.10 s^−1^ for an optimized nanoparticle catalyst performing CO_2_ hydrogenation to ethanol at 200°C [52]. In addition, the experimental data suggest that the TOF for CO_2_ hydrogenation to CH_4_ on optimized oxide‐supported Ru nanoparticles may reach ≈ 0.1 s^−1^ at T = 523.15 K [53], which is within the uncertainty of the estimated Sabatier optimum for CH_4_, corresponding to a TOF of 3.08 s^−1^ at T = 523.15 K (RMSE = ≈ 10^2^ times). Therefore, the close quantitative agreement between the microkinetic simulations, the TOFs estimated by our algorithm, and the available experimental data supports both the validity and the practical relevance of the present computational methodology.

### 2.5 Implications for Catalyst Design

The detailed analysis of the pathways for the formation of C2 oxygenates and the unwanted CH_4_ byproduct discussed in Note S7 indicates that the design of catalysts for CO_2_ hydrogenation should not be reduced to the stabilization of one key intermediate or to the optimization of one elementary step. Instead, efficient C2 oxygenate formation requires a balance between the formation of CH_x_ species, sufficient C–C coupling activity after CH_x_ formation, and efficient protonation of alcoholate and carboxylate intermediates. At the same time, certain highly stable intermediates, such as CO*, formate, acetate, or OH*, should not bind to the catalyst too strongly to avoid their excessive build‐up on the surface. Moreover, the rate of hydrogenation steps should not be too high to avoid undesired complete hydrogenation of CO_2_ into methane.

The need to balance these features is in line with the selection of Δ*E_ads_* (OH) and Δ*E_ads_* (CO) as the most indicative descriptors for the analysis of the two‐dimensional Sabatier volcanoes. Δ*E_ads_* (CO) was used as a proxy for the stability of C‐bound intermediates and the propensity for CO poisoning, whereas Δ*E_ads_* (OH) was used as a proxy for oxophilicity (i.e., stabilization of O‐bound intermediates) and C–O cleavage kinetics. Thus, the obtained volcano plots map the competing requirements in the CO_2_ hydrogenation network for the design of efficient catalysts.

The metal‐specific CO_2_ hydrogenation mechanisms detailed in Note S7 illustrate how deviations from this balance undermine the selectivity of various transition metals toward the formation of C2 oxygenates. On Au, the reaction was limited by the weak stabilization of surface intermediates: the clean surface remained the TDI, whereas the dissociation of CH_2_OH* into CH_2_* and OH* was identified as the TDTS. Thus, the network was limited by the inability of Au to form the CH_x_ pool required for further carbon chain growth. Cu was also located on the weakly binding side of the volcano and featured the same TDTS. In turn, the C–C coupling of CH_2_* with CO_2_*, CO*, or CH_2_O* was calculated to be kinetically feasible on Cu nanoparticles once CH_2_* had been formed. This explains why Cu sites may be suitable for catalysis of C–C coupling steps but insufficient to catalyze all steps in the CO_2_‐to‐C2 oxygenate pathway unless synergistically combined with other catalyst components facilitating CH_x_ formation [29].

More strongly binding metals exhibited other limiting features in the identified mechanisms. On fcc‐Co, the formate pathway was kinetically preferred over CO_2_ activation via the RWGS pathway, but the high barrier for HCOO* protonation and the high stability of HCOO* (or CH_3_COO*) intermediates increased the energetic span of the corresponding C_2_ oxygenate pathways. Therefore, the strength of Co−O interactions was beneficial for C−O cleavage and CH_x_ formation, but excessive stabilization of carboxylate species on Co surface reduced turnover in CO_2_ hydrogenation. On Pd and Pt, CO* becomes the most abundant reaction intermediate after CO_2_ activation via the RWGS pathway due to the strongly binding nature of these metals. In these cases, the key bottleneck in CO_2_ hydrogenation is the need to escape a CO‐poisoned surface state. In turn, Ni occupied an intermediate position between Co, Pd, and Pt due to its higher oxophilicity. On Ni, hydrogenation of HCOOH* to OCH_2_OH* became the TDTS, while CH_2_* or the clean surface was assigned as the TDI, depending on the product.

Rh was the closest monometallic system to the Sabatier optimum of CO_2_ hydrogenation into C2 oxygenates in Figure 5 because it avoided some of the kinetic chokepoints limiting the previously discussed catalysts. CH_x_ formation on Rh is more accessible than on Au and Cu, whereas the affinity to CO is weaker compared to Pd and Pt. At the same time, Rh was not predicted to be the perfect catalyst for the formation of C2 oxygenates. The same formate‐derived pool of CH_x_ intermediates that enabled access to C2 pathways in the reaction network could also be used to produce methane at a rate exceeding C–C bond formation. Therefore, Rh should be viewed as the best monometallic reference point in the considered material space, since our analysis indicates that catalysts with optimized CO* and OH* binding energies may achieve much higher activity and selectivity in CO_2_ hydrogenation.

The observed complexity of BEP relationships provides another catalyst design implication. Although step‐specific BEP relationships could be formulated in some cases, their slopes differed significantly even for C‐O cleavage in homologous intermediates such as OCH_2_OH* and CH_3_CH(O)OH*. For several C–C coupling steps, even step‐specific correlations did not yield a reliable BEP linear relationship between Δ*G_rxn_* and Δ*G* ^‡^. Importantly, such poor quality of linear BEP relationships could undermine common assumptions lying at the foundation of computational catalyst design methodology, for example, when evaluating competition between CO_2_ hydrogenation into methane and C2 oxygenates. Instead, our analysis suggests that a rigorous treatment of catalytic networks of such complexity is required to draw reliable conclusions.

Thus, our computational analysis suggests that the design of advanced catalysts for CO_2_ hydrogenation to C2 oxygenates should focus on the exploration of multicomponent or multimetallic catalysts, in which distinct sites catalyze various reaction steps while avoiding the kinetic bottlenecks identified above. For example, a more oxophilic metal in the catalyst composition may facilitate CO_2_ activation and C–O cleavage leading to CH_x_ formation. In turn, the presence of Cu or a metal with similar properties in the catalyst may promote C−C coupling at a faster rate than methanation. Finally, promoters and supports, among other effects, may help to suppress poisoning of the catalyst by CO, carboxylates, or alcoholates via spillover and facilitation of protonation steps.

## 3 Conclusions

This study proposes a new approach for the construction of Sabatier volcanoes for reactions with complex mechanisms and applies it to the analysis of CO_2_ hydrogenation toward methane and C2 oxygenates (acetic acid, ethanal, and ethanol) on Au, fcc‐Co, Cu, Ni, Pd, Pt, and Rh. The comprehensive analysis of a complete reaction network was achieved using the Pathfinder‐like algorithm and the energetic span model. This analysis showed that product‐specific activity trends correlate with surface binding energies of CO* and OH*, which reflect the balance between the stabilization of C‐bound and O‐bound intermediates in the CO_2_ hydrogenation network.

Moreover, our analysis demonstrated the limited transferability and insufficient accuracy of conventional linear BEP‐type relationships. Therefore, a NN model was introduced to account for reaction energies, metal identity, transition state classes, and reactant and product structures encoded with Coulomb matrices. This non‐linear NN model, designed for low‐data regimes and incorporating a convolutional architecture with an attention mechanism, achieved a substantially lower cross‐validated MAE than the linear BEP‐like model (≈ 0.23 vs. ≈ 0.36 eV) and higher out‐of‐sample accuracy.

All monometallic particles in our study were found to provide insufficiently balanced activity for efficient C2 oxygenate formation. CO_2_ hydrogenation on Au and Cu was calculated to be unfavorable due to kinetically hampered CH_x_ formation, on Co due to excessive stabilization of O‐bound intermediates, on Pd and Pt due to overstabilization of CO*, and on Rh due to preferential methanation of CH_x_ species. Although Rh was found to exhibit the highest production rate of C2 oxygenates among all considered metals, our analysis indicates that much more active and selective CO_2_ hydrogenation can be achieved on catalysts with optimized binding energies of OH* and CO* intermediates. Moreover, we conclude that catalyst design efforts should focus on the exploration of multicomponent systems or alloy nanoparticles with components that facilitate CO_2_ activation, CH_x_ formation, and efficient C–C coupling instead of methanation, while avoiding poisoning by highly stable intermediates. The obtained insights illustrate the power of the developed approach to model complex reaction networks and to provide practical recommendations for the design of advanced catalysts, e.g., for CO_2_ hydrogenation toward the sustainable production of bulk and platform chemicals.

## 4 Computational Details

VASP 6.3.2 was used to perform spin‐polarized DFT calculations employing the revPBE [54] functional. A plane‐wave energy cutoff at 415 eV and the PAW method were used [55]. The DFT‐D3 scheme with Becke‐Johnson damping was used to account for dispersion interactions [56, 57].

Microkinetic simulations were conducted using the MKMCXX program [58].

SciPy [59], NumPy [60], scikit‐learn [61], Pandas [62], and NetworkX [63] Python libraries were used routinely in the underlying code. All neural‐network architectures were built and trained in TensorFlow using the Keras API [64]. DScribe library [65] was used for fast calculation of Coulomb matrices. Plotly library and Avogadro [66] were used for plotting and atomistic model visualization, respectively.

Grok4 and ChatGPT‐5.1 were utilized for initial text proofreading and code refactoring.

## Author Contributions

**Mikhail V. Polynski**: conceptualization, formal analysis, investigation, methodology, writing – original draft, writing – review and editing, software, visualization. **Sergey M. Kozlov**: conceptualization, funding acquisition, writing – review and editing, resources.

## Conflicts of Interest

The authors declare no conflicts of interest.

## Supporting information




**Supporting File 1**: advs75932‐sup‐0001‐SuppMat.pdf.

**Supporting File 2**: advs75932‐sup‐0002‐TableSI15.xlsx.

**Supporting File 3**: advs75932‐sup‐0003‐SI7.zip.

## Data Availability

The data that supports the findings of this study are available in the supplementary material of this article and in a dedicated GitHub repository [67].
