# Supplementary material for: Construction of Sabatier Volcanoes for CO2 Hydrogenation to C1‐2 Oxygenates Using Data‐Efficient Machine Learning
Source: Adv Sci (Weinh). 2026 Jun 16:e75932. Online ahead of print. doi: 10.1002/advs.75932 (PMC13336856; doi:10.1002/advs.75932)
Supplement: Supplementary file 1 — Supporting File 1: advs75932‐sup‐0001‐SuppMat.pdf. [file ADVS-9999-e75932-s002.pdf]

Supporting Information for the Article

Construction of Sabatier Volcanoes for CO_2 Hydrogenation to C1-2 Oxygenates Using Data-Efficient Machine Learning

Mikhail V. Polynski,[1], Sergey M. Kozlov[1]*

[1] Department of Chemical and Biomolecular Engineering, National University of Singapore, 4 Engineering Drive 4, Singapore 117585, Singapore.

Corresponding Authors

* (S.M.K.) sergey.kozlov@nus.edu.sg

Table of Contents: Supplementary Notes

S1. DFT Calculations: Computational Details S3
S2. Remarks on Observed BEP Relationships S5
S3. Machine Learning: Computational Details S7
S4. Estimating Catalytic Activity: Model and Code Workflow S17
S5. Descriptor Selection for Sabatier Volcano Plots S24
S6. Additional Discussion of Sabatier Volcano Plots S29
S7. Representative Mechanisms and Estimated Activities S31
S8. Microkinetic Simulations S43
S9. References S45

S1. DFT Calculations: Computational Details

Spin-polarized DFT calculations were carried out in VASP 6.3.2[1] using the revPBE functional,[2] selected for its suitability for calculations of metallic systems (unlike hybrid functionals)[3] and exceptional accuracy among other GGA functionals.[4] A plane-wave cutoff energy was set to 415 eV; core-valence interactions were treated using the PAW method.[5] Dispersion interactions were incorporated via the DFT-D3 scheme with Becke–Johnson damping.[6,7] Brillouin zone sampling was limited to the Γ-point because our study considered only nanoparticle models, and electronic occupancies were defined using the Fermi-Dirac function with a smearing parameter equal to 0.03 eV. Energy convergence thresholds were set to the following values: 10^{-5} eV for structural relaxations, 10^{-6} eV for TS structure optimizations, and 10^{-7} eV for vibrational frequency analyses. Each KS-SCF cycle contained up to 400 electronic steps. Real-space projection of wavefunctions was disabled (LREAL = .FALSE.) to increase the numerical quality of the calculated potential energy surface (PES). All symmetry constraints were removed (ISYM = −1) to ensure unbiased structural relaxation. Non-spherical contributions to the gradient corrections within the PAW formalism (LASPH = .TRUE.) were included, which is critical for accurately modeling transition metal systems.

All systems were constructed in orthorhombic simulation cells with cell vectors guaranteeing a minimum vacuum spacing of 10.0 Å between periodic images of M_{79} (M = Au, Co, Ni, Pt, Rh) nanoparticles. The geometry optimization procedures for transition states on Au, Pt, and Rh were initiated from the corresponding TS structures optimized on Pd_{79} NPs in our previous work,[8] whereas TS optimized on Cu_{79} were used to initiate the calculations on Ni and fcc-Co NPs. Transition states were re-optimized using the DIMER method, invoking the FIRE optimization algorithm[9] from the VTST suite,[10–13] employing a force convergence threshold of 0.03 eV $Å^{-1}$.

The reaction coordinate for the DIMER optimizations was set using the MODECAR file from the corresponding TS on Pd or Cu. Each optimized transition state featured a single imaginary frequency aligned with the reaction coordinate, as verified by vibrational analysis of non-metal atoms.

Geometry optimizations of states corresponding to PES minima were carried out using the FIRE algorithm or, if convergence failed, the L-BFGS method from the VTST Tools, applying a force threshold of 0.01 eV $Å^{-1}$. To obtain initial and final state structures, TS geometries were perturbed in both directions along the reaction coordinate. In rare cases, low-magnitude imaginary frequencies (<50i cm^{-1}) were observed in PES minima, attributable to hindered translational or rotational motions of weakly adsorbed species such as CH_4 or CO_2. These imaginary modes were treated as real modes by taking the absolute value of the imaginary number in thermochemical calculations, consistent with the stringent convergence thresholds applied.

Thermochemical corrections were calculated using the ASE[14] Thermo module. For nanoparticle systems, only harmonic vibrational contributions were included. Gas-phase species were treated within the ideal gas, rigid rotor, and harmonic oscillator approximations. All thermochemical calculations were performed at two sets of conditions: 1) standard IUPAC conditions (1 bar, 273.15 K) and 2) reaction conditions common for the synthesis of C_{2+} oxygenates via CO_2 hydrogenation: temperature T = 523.15 K, p(H_2) = 30 bar, p(CO_2) = 10 bar.[15] Partial pressures of stable intermediates and products were chosen as 1 bar, representing a simplified approximation of their low but nonzero concentrations during steady-state operation. In the second case, we retained the harmonic vibrational corrections evaluated at 273.15 K for surface-adsorbed states to avoid the unphysically large vibrational entropy contributions that arise in the harmonic approximation at elevated temperatures, when anharmonicity becomes significant.

S2. Remarks on Observed BEP Relationships

Section 2.2 in the main text examines C–C coupling steps proceeding via metathesis-like transition states (Figure 2d in the main text), where no clear linear Brønsted–Evans–Polanyi (BEP) trend was observed due to substantial scatter in the data. Although a similarly high RMSE of BEP relations was also observed for C–O bond cleavage steps (Figure 2a), in the latter case, strong linear BEP correlations were recovered when the analysis was performed individually for each elementary step (Figure 2b). However, as shown in Figure S1, the same approach does not yield higher-quality BEP relationships for C–C coupling steps. For example, no linear correlation is observed between the activation free energies, $\Delta G^{\ddagger}$, and the respective reaction free energies, ΔG_{rxn}, for the following elementary steps (Figure S1):

$$CH_2{}^* + CO^* \longrightarrow CH_2CO^* \quad (1),$$

$$CH_2{}^* + CO_2{}^* \longrightarrow CH_2COO^* \quad (2).$$

Combined with the additional examples discussed in Section 2.2, this suggests that classical BEP relationships (i.e., $\Delta G^{\ddagger}$ as a function of ΔG_{rxn} alone in a set of structurally similar transformations[16]) may fail to hold for some elementary steps. Instead, metal identity and structural features of the reactants and products may play an equally important role in determining activation barriers for elementary steps within branched reaction networks.

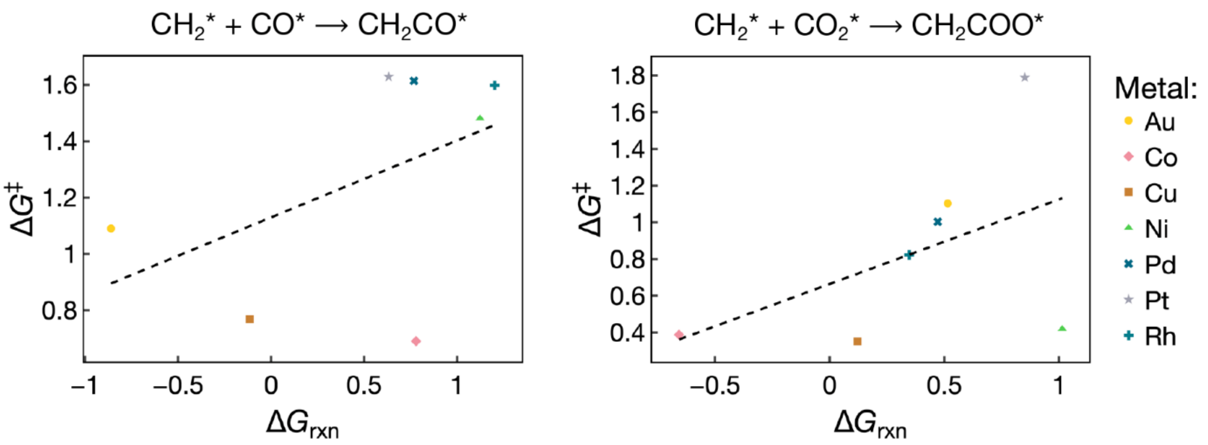

Figure S1. $\Delta G^{\ddagger}$ in elementary steps (1) and (2) plotted against the corresponding ΔG_{rxn}.

Table S1. Parameters[a] of Brønsted-Evans-Polanyi relationships for representative classes of elementary steps in the CO_2 hydrogenation network. The first six entries in the table correspond to the data shown in Figure 2 of the main text.

Class of elementary steps	Slope	Intercept	RMSE	MAE
C–O cleavage, reversed nucleophilic addition (Figure 2a)	0.46	0.72	0.39	0.33
C–O cleavage in OCH_2OH^* (Figure 2b, purple line)	0.17	0.36	0.09	0.07
C–O cleavage in $CH_3CH(O)OH^*$ (Figure 2b, magenta line)	0.55	0.14	0.06	0.06
Homolytic C–O bond cleavage (Figure 2c)	0.22	1.81	0.15	0.13
C–C coupling involving CH_2 and CH species (Figure 2d, black line)	0.36	1.00	0.40	0.33
C–C coupling involving addition of CH_3^* (Figure 2e)	0.32	1.62	0.20	0.16
$CH_2^* + CO^* \longrightarrow CH_2CO^*$ (Figure S1, left)	0.27	1.13	0.33	0.28
$CH_2^* + CO_2^* \longrightarrow CH_2COO^*$ (Figure S1, right)	0.46	0.66	0.42	0.31

[a] Intercept, RMSE, and MAE values are in eV; slope and R^2 values are dimensionless.

S3. Machine Learning: Computational Details

We encoded metal identity (Au, fcc-Co, Cu, Ni, Pd, Pt, Rh) and transition state class via one-hot vectors, with seven binary indicator variables for metals and seven for transition state types. The elementary steps were divided in the following way to represent TS classes (see detailed description in a prior work[8]):

1. Addition to C(sp^2) in hydrogenation steps;
2. C(sp^3)-like TS in hydrogenation steps;
3. Protonation leading to the formation of OH groups;
4. Protonation leading to the formation of COOH groups;
5. "Metathesis-like" C–C coupling;
6. C–C coupling by CH_3 addition to C=Z (Z = O, C);
7. C–O cleavage yielding OH and an intermediate having a C=Z fragment (Z = O, C).

We appended the reaction free energy, ΔG_{rxn}, as a continuous feature, yielding a 15-dimensional descriptor vector for each elementary step. To create the hold-out set, we used StratifiedShuffleSplit (n_splits = 1, test_size = 38, random_state = 42) of scikit-learn, stratifying on the original TS class labels to preserve their relative frequencies. This produced a fixed hold-out partition of 38 entries and a training pool of 177 entries. We then reused the very same holdout set to guarantee consistent evaluation across methods.

Ridge regression. For our baseline "generally linear" model, we applied the default Kernel Ridge Regression of scikit-learn with a linear kernel. We used GridSearchCV over $\alpha \in [10^{-3}, 10^{-2}, 10^{-1}, 1, 10]$ with 5-fold cross-validation. The procedure resulted in selecting $\alpha = 10^{-3}$ or 10^{-2} as optimal in the models for the prediction of forward and reverse barriers, respectively (Figure 3 in

the main text). We did not extend the search to smaller α in order to maintain sufficient regularization and avoid overfitting.

CatBoost. We next trained CatBoostRegressor[17] using L2 loss. We tuned four hyperparameters by 5-fold CV (Table S2).

Table S2. Hyperparameter grids for forward and reverse $\Delta G^{\ddagger}$ models.

Hyperparameter	Forward $\Delta G^{\ddagger}$ grid	Reverse $\Delta G^{\ddagger}$ grid
iterations	60, 70, 80	70, 80, 90, 100
learning_rate	0.20, 0.225, 0.25	0.25, 0.275, 0.30
depth	1, 2	1, 2
l2_leaf_reg	2.0, 3.0, 4.0	0.5, 1.0, 2.0

For the forward $\Delta G^{\ddagger}$ model, grid search identified depth = 1 ("generally linear" behavior of the approximated dependency), iterations = 70, learning_rate = 0.225, and l2_leaf_reg = 3.0 as optimal. For the reverse $\Delta G^{\ddagger}$ model, it selected depth = 1, iterations = 80, learning_rate = 0.275, and l2_leaf_reg = 1.0. We then refitted both final models on their respective full training sets and evaluated performance on the hold-out set via RMSE and MAE (see Figure S2).

We held all convergence criteria and random seeds (seed = 42) constant to ensure full reproducibility.

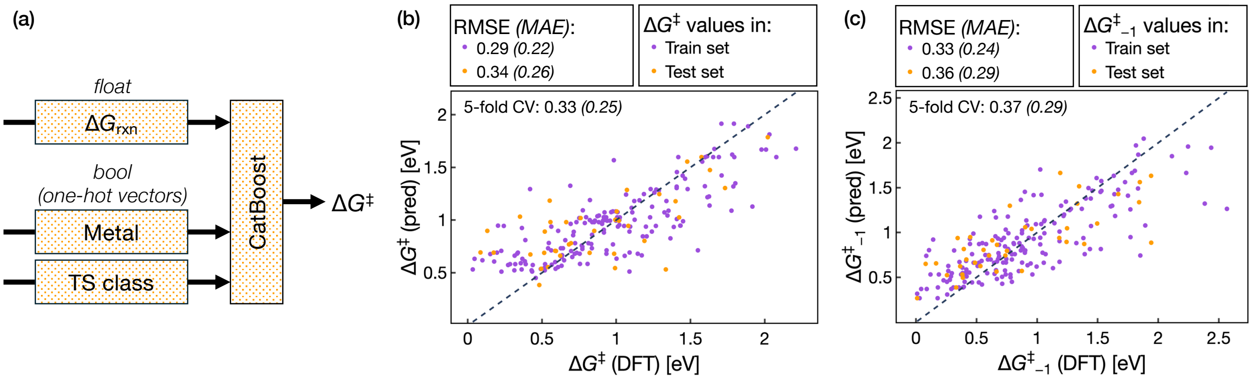

Figure S2. CatBoost regression model: (a) model features; (b) parity plot for $\Delta G^{\ddagger}$ values in forward steps; (c) parity plot for $\Delta G^{\ddagger}$ values in reverse steps.

Importantly, CatBoost regression did not yield any significant improvement over ridge regression. Gradient boosting methods are known for their robustness to unscaled input data, and we deliberately omitted autoscaling to preserve the physical meaning of all descriptors, so every feature used in the ridge and CatBoost models carries explicit physicochemical significance.

For our neural network models, we additionally incorporated Coulomb matrix elements, which also bear a form of physical interpretation. The obtained matrix elements can attain very large values, often tens to hundreds, when transition metals (or other heavier elements with $Z > 10$) are involved (see Figure 4a in the main text for definition). Note that the neural network model achieved clearly higher accuracy compared to the ridge regression, unlike the CatBoost approach, which was inherently tolerant to unscaled inputs. This observation suggests that parameter scaling would have had a marginal effect on model performance, so this technique was omitted in our study.

Neural network model. The final neural network model that we used for inference combines four parallel input branches and was trained with the following hyperparameters and protocol. First, we represented each elementary step by:

1. A 100×100 Coulomb-matrix interaction map was constructed by first computing 10×10 Coulomb matrices for the surface specie structures representing the reactant and product in an elementary step using DScribe,[18] flattening each matrix into a 100-dimensional vector, and taking the outer product of the two resulting vectors (Figure 4a);

2. A one-hot vector for metal identity (7D);

3. A one-hot vector for transition state class (7D);

4. The reaction free energy, ΔG_{rxn}, as a scalar/float.

The 100×100 interaction map was reshaped to 100×100×1 and passed through a Conv2D layer with 10 filters of size 10×10, strides of 10×10, and ReLU activation, yielding a 10×10×10 tensor. This tensor was processed by a squeeze-and-excitation attention block with a reduction ratio of 2, in which channel-wise weights were obtained by global average pooling followed by 5- and 10-neuron dense layers with ReLU and sigmoid activations, respectively. The reweighted convolutional tensor was then reduced by global average pooling to a 10-dimensional feature vector. In parallel, the one-hot metal identity and TS type vectors were processed through separate ReLU-activated dense layers, while ΔG_{rxn} was provided directly as a scalar input. These features were concatenated and passed through a 10-neuron hidden dense layer with ReLU activation, followed by a one-neuron dense output layer with softplus activation.

All convolutional and dense layers, except for the final output layer, used the default Glorot uniform kernel initializer. For the final softplus output layer, we applied the LeCun normal kernel initializer, which was necessary to keep the predictions numerically stable.

We trained this architecture in TensorFlow/Keras with the Adam optimizer (learning rate 1×10^{-3}), batch size 16, for up to 100 epochs, and used early stopping (patience 10) to monitor validation RMSE. 10% of the initial training set was outsourced to the validation set. To control for stochasticity in weight initialization and data ordering, we performed 20 independent runs and finally selected the single best-performing ML model for inference. Table S3 below includes the average accuracy metrics of the NN models obtained in these 20 independent training runs. Model architecture is visualized in Figure S3 below.

Table S3. Average accuracy of the NN models for activation free energies, $\Delta G^{\ddagger}$, in forward and reverse steps on the training and holdout sets, as well as in 5-fold cross-validation.

	Forward $\Delta G^{\ddagger}$ [eV]	Reverse $\Delta G^{\ddagger}$ [eV]
Training	0.26 ± 0.03 (0.20 ± 0.03)*	0.26 ± 0.03 (0.20 ± 0.02)
Holdout	0.30 ± 0.04 (0.24 ± 0.02)	0.35 ± 0.02 (0.28 ± 0.02)
5-fold CV (average)	0.30 ± 0.01 (0.23 ± 0.01)	0.33 ± 0.02 (0.25 ± 0.02)

* RMSE and MAE (in parentheses) values are in eV. Mean RMSE (MAE) ± standard deviation from the mean RMSE (MAE) over 20 trained models is shown in every column.

To assess the contribution of the attention mechanism, we built a no-attention variant by removing only the squeeze-and-excitation block from the Conv2D branch; all other layers and training protocols were kept identical (Figure S4). Table S4 compares the performance of this variant with that of the full model. A Welch two-sample *t*-test applied to the RMSE values from 20 independent runs showed that the full model achieved significantly lower errors on the holdout set ($t = -2.38, p = 0.024$) and in 5-fold cross-validation ($t = -2.70, p = 0.011$). These results indicate that the attention mechanism improves model accuracy and generalizability. In addition, the standard deviation of the holdout RMSE across the 20 independent runs increased from 0.04 eV

with attention to 0.08 eV without attention, indicating reduced run-to-run robustness when the attention block was removed.

Table S4. Comparison of the full model (includes attention and Coulomb matrix / Conv2D blocks) *vs.* the model lacking the attention mechanism vs. the "simplistic" model (no Conv2D, no attention).

Model	Training RMSE*	Validation RMSE	Holdout RMSE	CV RMSE
Full (with attn.)	0.26 ± 0.03	0.28 ± 0.05	0.30 ± 0.04	0.30 ± 0.01
No-attention	0.30 ± 0.08	0.30 ± 0.04	0.33 ± 0.08	0.31 ± 0.02
Simplistic	0.28 ± 0.02	0.30 ± 0.05	0.33 ± 0.03	0.32 ± 0.004

* Mean RMSE ± standard deviation from the mean RMSE (over 20 trained models) is shown in every column; error bars therefore reflect variability between trainings of the *same architecture, not uncertainty of the mean*. All values are in eV.

We also trained a "simplistic" model that uses exactly the same input features as the kernel-ridge and CatBoost regressors by deleting the entire Coulomb matrix branch (no Conv2D, no attention, Figure S5). This model shares the same downstream dense layers and softplus output as the full NN. Its performance is summarized in Table S4. Welch two-sample *t*-tests applied to the RMSE values from 20 independent runs showed that the full architecture featuring Coulomb matrixes, convolution, and attention mechanism achieved significantly lower errors than the simplified variant in both 5-fold cross-validation ($t = -7.34$, $p = 8.09 \times 10^{-8}$) and on the holdout set ($t = -3.14$, p = 3.46×10^{-3}). These results indicate that the Coulomb matrix interaction branch improves out-of-sample predictive performance beyond the tabular descriptors alone.

For inference on reductive elementary steps (hydrogenation and protonation steps; see Section 2.3 in the main text), we fed the pre-computed Coulomb matrices, one-hot encoded metal and TS

class, and ΔG_{rxn} into the single best NN instance (selected by the lowest RMSE in the CV, as well as on testing and holdout sets), obtaining $\Delta G^{\ddagger}$ predictions directly from the output layer.

The NN model for predicting reverse activation barriers was trained using the corresponding ΔG_{rxn} values of the reverse steps, with product and reactant structures swapped relative to the forward case, while keeping all other input features the same.

Finally, for completeness, Table S5 lists the performance metrics on the validation set for the final neural network models used for inference.

Table S5. Performance of final NN models (selected for inference) on the validation set.

	Forward $\Delta G^{\ddagger}$ [eV]	Reverse $\Delta G^{\ddagger}$ [eV]
Validation	0.30 (0.24)*	0.24 (0.20)

* RMSE and MAE (in parentheses) values are in eV.

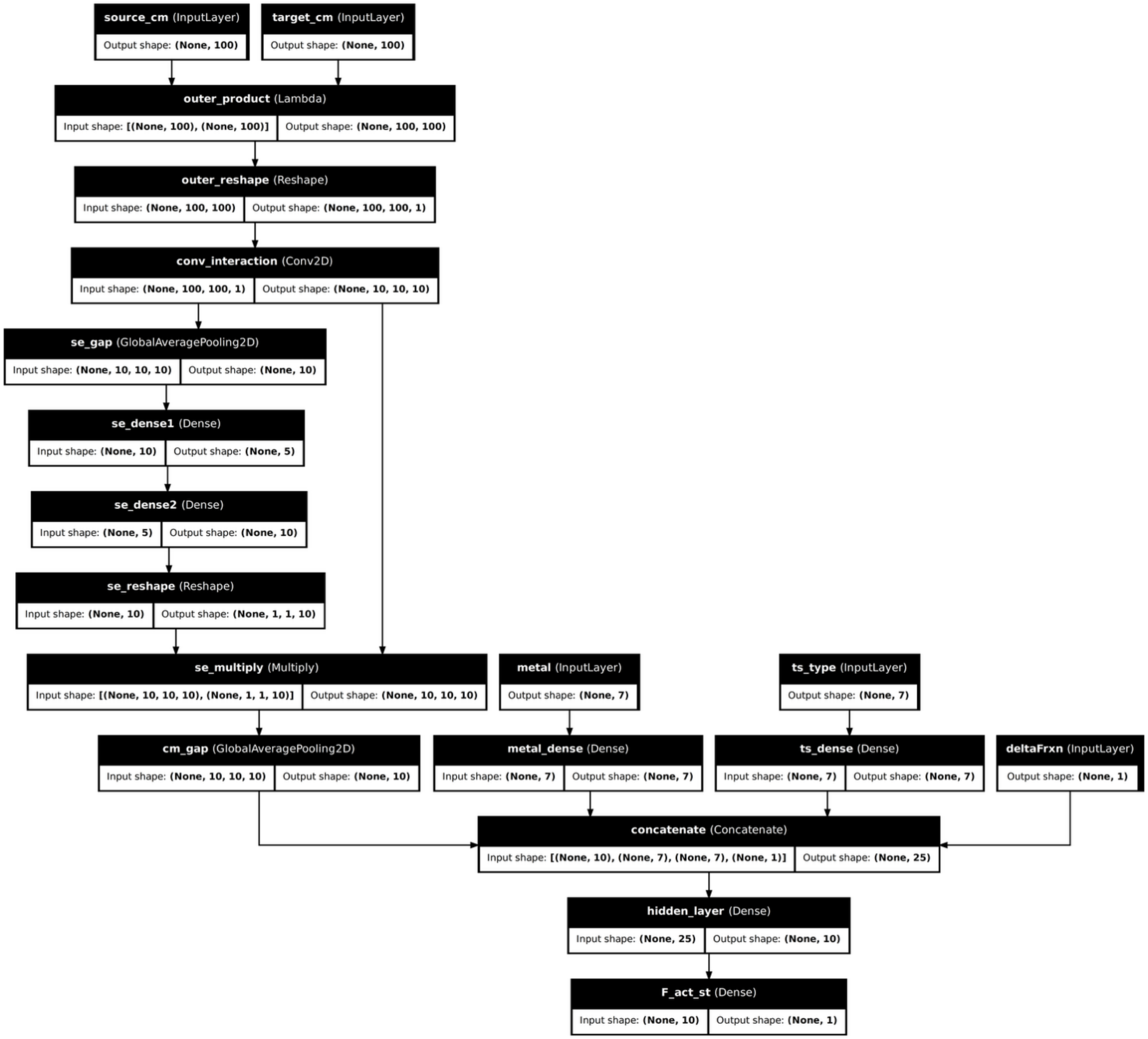

Figure S3. Main model: the architecture of the convolutional neural network featuring the attention mechanism used to predict activation free energies ($\Delta G^{\ddagger}$). This model architecture was used to generate the results discussed in the main text.

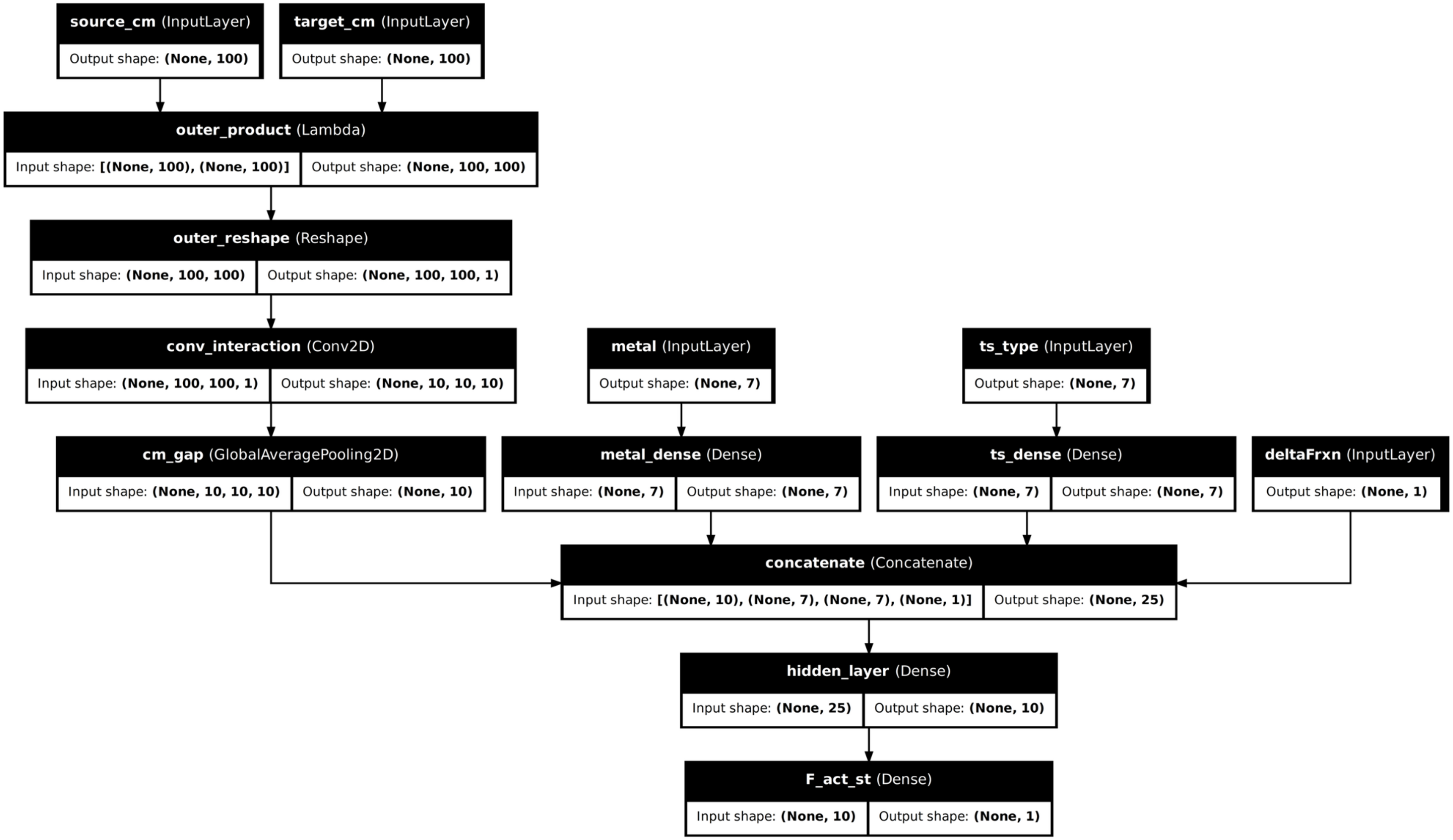

Figure S4. "No attention" model, which was used for accuracy benchmarking only.

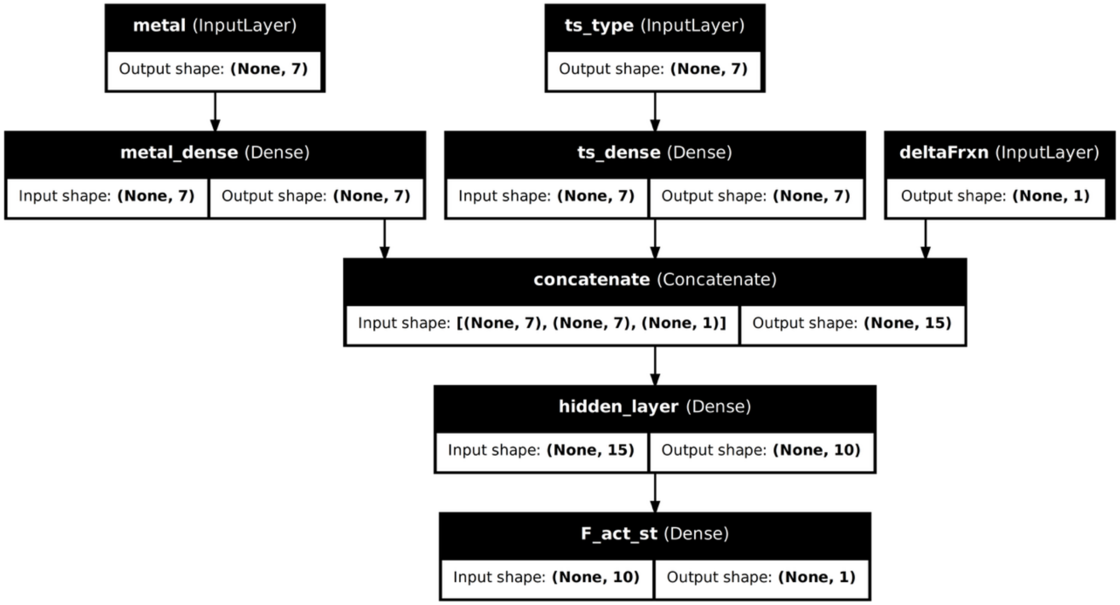

Figure S5. Simplified model lacking the whole Coulomb matrix branch, which was used for accuracy benchmarking only.

S4. Estimating Catalytic Activity: Model and Code Workflow

Estimating TOF. Equation (20) in the article by Kozuch[19] expresses the TOF of a catalytic network in the *E*-representation:

$$\mathrm{TOF} = \frac{k_\mathrm{B}T}{h} \cdot \frac{(\sum_n \mu_n)(1 - e^{\Delta G_r/RT})}{(\sum_i \tau_i)(\sum_j e^{(I_j + \delta G_{ij})/RT})},$$

The description of each term in this equation is given below:

n labels the different mechanisms embedded in the catalytic network (that is, single-cycle subgraphs);

i labels the spanning trees of the corresponding reaction graph;

j enumerates the intermediates;

$\mu_n = e^{-\sum_{k \in n} T_k/RT}$ is the exponential of the sum of the transition state energies of n-th mechanism, divided by RT;

$\tau_i = e^{-\sum_{k \in i} T_i/RT}$ is the exponential of the sum of the transition state energies of the i-th spanning tree, divided by RT;

I_j is the Gibbs free energy of the j-th intermediate;

$\delta G_{ij} = \Delta G_r$ if, within spanning tree i, the transition state that closes the catalytic cycle precedes intermediate j, and is 0 otherwise;

ΔG_r being the reaction Gibbs free energy;

k_B, h, and R are Boltzmann, Planck, and universal gas constants, respectively.

The total TOF can be split between mechanisms producing the *same* product (here, CO, HCOOH, HCHO, EtOH, etc.). E.g., for EtOH, we can write:

$$\mathrm{TOF(EtOH)} = \sum_n \mathrm{TOF}_n,$$

With n labelling one out of M mechanisms of EtOH production. In our case, the reaction network represents the formation of CH_4, CH_3COOH, CH_3CHO, and EtOH in a labeled digraph:

$$Graph = (Vertices, Edges),$$

Where vertices carry weights ΔG_n (Gibbs free energies of intermediates relative to isolated gas-phase CO_2 and H_2); *directed* edges carry either forward or reverse free activation energies, $\Delta G^{\ddagger}$, as the weights. In such a way, for every defined gas-phase intermediate or product, we have *M mechanisms* (closed walks) going from the state of gaseous CO_2 (and $1/2H_2$ implied due to the presence of hydrogenation and protonation steps) over the clear catalyst surface to the adsorbed product, whose desorption energy ΔG_{des} is later accounted for in ΔG_r.

The key assumption in the ansatz proposed for the ***sampling*** *of catalytic activity is that the catalytic activity is governed by the mechanism with the highest TOF_n.* Indeed, smaller-TOF_k mechanisms should offer exponentially small contributions to the total TOF. By rearranging the initial equation and focusing only on a single mechanism, we obtain:

$$\mathrm{TOF}_n = \frac{k_\mathrm{B}T}{h} \cdot \frac{(1-e^{\Delta G_r/RT})}{(\frac{\sum_i \tau_i}{\mu_n})(\sum_j e^{(I_j+\delta G_{ij})/RT})}.$$

The latter equation can be simplified to the conventional energetic span model for a specific mechanism:[20]

$$\mathrm{TOF}_n = \frac{k_\mathrm{B}T}{h} \cdot \frac{e^{-\Delta G_r/RT}-1}{(\sum_{i,j=1}^{N} e^{(T_i-I_j-\delta G_{ij})/RT})} \qquad (1),$$

Here, T_i and I_j are Gibbs energies of transition states and intermediates within this mechanism, respectively; $\delta G_{ij} = \Delta G_r$, if the *i*-th TS comes after the *j*-th intermediate, or zero otherwise.

In summary, we propose to use so-defined TOF_n as the metric of catalytic activity in (high-throughput) computational screening for active catalysts. Importantly, TOF_n values are positive for $\Delta G_r < 0$, which implies the presence of thermodynamic driving force for the reaction to

proceed in the forward direction. Under the considered reaction conditions, this requirement is satisfied for CO_2 hydrogenation into CH_4, CH_3COOH, CH_3CHO, and EtOH, which are the main products considered in this work. Note that other reaction conditions would be required to satisfy the $\Delta G_r < 0$ requirement for other possible CO_2 hydrogenation products.

Predicting the most probable mechanisms. To identify, within the extended reaction network, those catalytic cycles that are most likely to control the turnover for a given product, one can use the graph-based PATHFINDER algorithm of Türtscher and Reiher.[21] In its original formulation, PATHFINDER encodes a chemical reaction network as a bipartite graph, with distinct vertices for compounds and reactions. Edges connect compounds to reaction vertices and *vice versa* and carry both kinetic and stoichiometric information in the form of activation barriers and lists of required reagents. Free energies of activation are converted to rate constants through Eyring's theory and are normalized to relative rates, which are then transformed by a decreasing cost function so that fast, low-barrier steps receive smaller kinetic weights. In addition, a set of iteratively determined "compound costs" quantifies the difficulty of forming each species from chosen starting compounds. Starting from finite costs for the feed compounds and effectively infinite costs for all others, PATHFINDER searches for shortest paths through the weighted graph and updates the compound costs until self-consistency is reached. The final edge weights combine kinetic accessibility and reagent demand, and shortest-path algorithms can then be used to extract likely reaction channels between any pair of compounds. This approach is particularly attractive for large reaction networks where explicit microkinetic simulations would be impractical or technically difficult. Importantly, path weights are best interpreted as relative probabilities rather than exact TOFs.

We applied a PATHFINDER-type approach to identify catalytic cycles that dominate the turnover for a given CO_2 hydrogenation product on each metal. Namely, we generated a compact set of kinetically favorable, stoichiometrically feasible reaction sequences connecting the initial state, gaseous CO_2 over the clean nanoparticle surface, to a given product. This allowed us to estimate the reaction rate towards the considered product via the energetic span model (equation 1) applied to the most kinetically favorable (most relatively probable) mechanistic pathway. In other words, PATHFINDER provided the ranking of candidate mechanisms in terms of relative probability and determined the sequence of intermediates involved, whereas the energetic span model is used to estimate relative TOFs of these candidates.

Our implementation follows the spirit of PATHFINDER and the energetic span model. Each metal-specific reaction network is represented as a digraph in which most vertices correspond to surface intermediates (while there are additional nodes for gas-phase species: H_2, CO_2, CO, HCOOH, CH_2O, CH_3OH, H_2O, CH_4, CH_3COOH, CH_3CHO, CH_3CH_2OH) and the directed edges correspond to elementary steps between them (thermally activated steps or adsorption/desorption). Every vertex carries the Gibbs free energy ΔG_i relative to gaseous H_2, CO_2, and H_2O over the clean catalyst surface. Each edge from node i to node j representing a thermally activated step carries the free energy of activation, $\Delta G^{\ddagger}_{i \to j}$ (activation energies of reverse steps, $j \longrightarrow i$, are also included). Adsorption/desorption edges carry reaction free energy, $\Delta G_{i \to j}$. Furthermore, each edge is annotated with a list of "required" intermediates that must be present on the surface or in the gas phase for the corresponding step to proceed. For example, hydrogenation steps require H* as a co-reactant, while C–C coupling steps require the appropriate C_1 partner such as CO*, CH_2O*, or CO_2*. This representation maps the original bipartite compound-reaction graph into a simpler

digraph of intermediates, which is sufficient for the catalytic cycles considered here while still retaining the essential energetic and stoichiometric information.

Kinetic edge weights are then assigned in close analogy to the PATHFINDER approach. For each elementary step i, we compute an Eyring rate constant k from $\Delta G^{\ddagger}_{i \rightarrow j}$ at the chosen temperature and normalize over all steps in the network to obtain relative rate constants:

$$p_i = \frac{k_i}{\sum_j k_j}.$$

The kinetic contribution to the edge weight is defined as:

$$w_i^{kin} = -\ln (p_i),$$

So that low-barrier, fast steps correspond to small weights and are favored in shortest / lowest cost path searches. To encode the availability of reactants (reflecting here surface concentration or gas pressure), we use compound costs c_i that quantify the difficulty of forming each intermediate from selected feed species. Analogously to PATHFINDER, we initialize c_i with finite values for the nodes representing the feed compounds and with effectively infinite values for all other nodes, and we then iterate a procedure. In each iteration, we search for the shortest paths from each feed node to all other nodes using dynamic edge weights that add w_i^{kin} to the sum of compound costs, c_i, of the intermediates listed as "required" on that edge (excluding the current source intermediate to avoid double counting), accumulate the corresponding path weights, and update c_i whenever an optimal path is found. Once the compound costs have converged, we set the final PATHFINDER-type edge weight for an elementary step i to:

$$w_i = w_i^{kin} + \sum_j c_i,$$

Where the sum runs over all required intermediates on that edge. The numerical values used for the feed-compound costs reflect the imposed initial gas pressures and are specified below.

Finally, for each target adsorbed product, we generate mechanistic candidates by computing the shortest simple paths to the corresponding product node in the weighted graph and retaining only those paths whose total cost lies within a small tolerance of the minimal value. For every candidate cycle, we then evaluate TOF using Equation (1). The mechanism with the lowest cost for a given product is used as the representative mechanism for constructing the Sabatier volcano (see below) and comparing catalytic activities across metals, whereas alternative near-degenerate cycles can be used for qualitative mechanistic discussion.

Code workflow and computational details. The initial reaction network, comprising 47 nodes and edges connecting them, was represented as a directed graph with nodes as intermediates (see Figure 1). The nodes representing HCOOH*, CH_2O*, CO*, CH_3OH*, CH_4*, CH_3COOH*, CH_3CHO*, and CH_3CH_2OH* had edges connecting them to the corresponding gaseous products and intermediates, as well as to CO_2, to close the catalytic cycles. Closure steps were included in the energetic span model calculations but disregarded in the revised PATHFINDER approach. The code calculates the lowest-cost pathways (i.e., most "probable," as per the original PATHFINDER formalism) and then calculates TOF. The reaction network was constructed from tabulated data using the NetworkX library,[22] defining CO_2* (node 1) as the reactant and adsorbed products such as HCOOH*, CO*, CH_3OH*, CH_4*, CH_3COOH*, CH_3CHO*, and CH_3CH_2OH* (nodes 4, 13, 7, 21, 41, 29, 33, 37 in the dedicated code and spreadsheets) as targets. Since these target states reflect the intermediates and products prior to desorption, ΔG_{des} of the product was accounted for in ΔG_r. Two sets of initial compound costs were used. The primary choice corresponds to feed gases, H_2 and CO_2, at pressures of 30 and 10 bar, respectively. In additional scenario, we retained the same pressures of H_2 and CO_2 and assigned initial finite costs to HCOOH*, CO*, CH_3OH*, CH_4*, CH_3COOH*, CH_3CHO*, and CH_3CH_2OH* corresponding to pressures of 1 bar to assess the effect

of reaction progression on the most favorable mechanism. Supplementary Note S7 below includes the discussion of the optimal reaction pathways, while the supplementary ZIP archive includes the underlying code.

S5. Descriptor Selection for Sabatier Volcano Plots

Descriptor selection is of utmost importance in the prediction of catalytic activity using Sabatier volcano plots. For the correlations we built in this work, we considered a set of descriptors representing the adsorption energies of basic species involved in the reaction mechanism. Initially, we considered $\Delta E_{ads}(\mathrm{CO})$, $\Delta E_{ads}(\mathrm{OH})$, $\Delta E_{ads}(0.5\mathrm{H_2})$, $\Delta E_{ads}(\mathrm{CH})$, $\Delta E_{ads}(\mathrm{CH_2})$, and $\Delta E_{ads}(\mathrm{CH_3})$ as potential descriptors. Note that dissociative adsorption was considered in the case of H_2. The initial selection was driven by the simplicity of the adsorbate composition, as well as the need to sample the strength of metal bonds with H, O, and C atoms. It was found that $\Delta E_{ads}(\mathrm{CH})$, $\Delta E_{ads}(\mathrm{CH_2})$, and $\Delta E_{ads}(\mathrm{CH_3})$ linearly correlate with each other, as Figure S6a below demonstrates. In addition, $\Delta E_{ads}(0.5\mathrm{H_2})$ was found to linearly correlate with $\Delta E_{ads}(\mathrm{CO})$, as seen from the data in Figure S6b. Therefore, only $\Delta E_{ads}(\mathrm{CO})$, $\Delta E_{ads}(\mathrm{OH})$, and $\Delta E_{ads}(\mathrm{CH})$ were considered in the final Sabatier volcano fits. Among possible combinations of these descriptors, $\Delta E_{ads}(\mathrm{CO})$ and $\Delta E_{ads}(\mathrm{OH})$ yielded the most accurate Sabatier volcano fits and resulted in physically meaningful fitting parameters (see below).

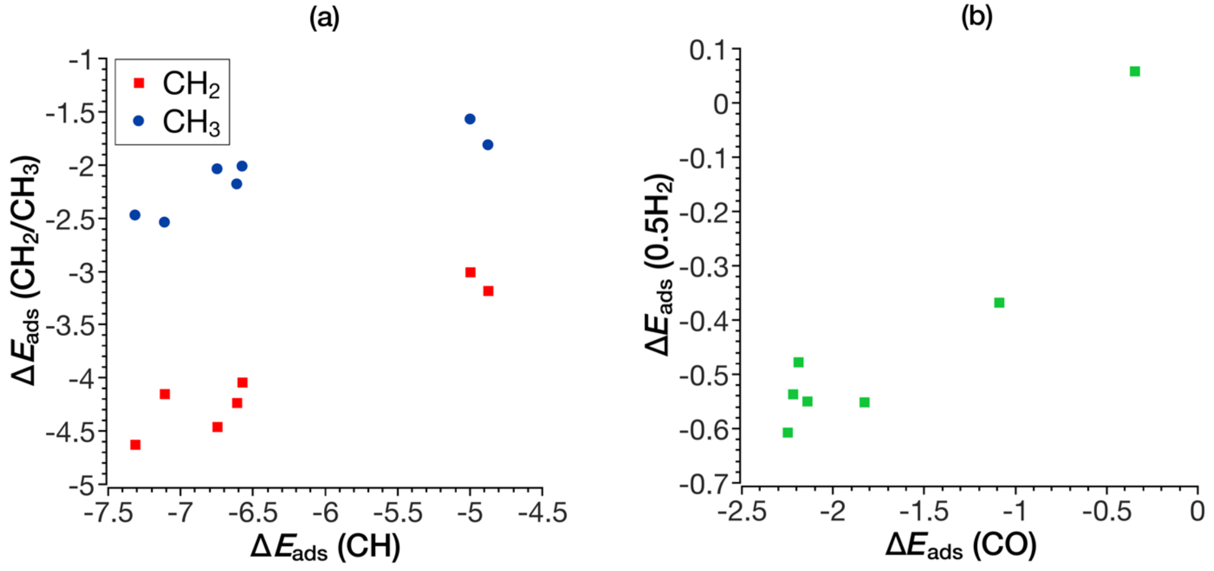

Figure S6. Linear correlation of descriptors: (a) $\Delta E_{ads}(\mathrm{CH})$ vs. $\Delta E_{ads}(\mathrm{CH_2})$ and $\Delta E_{ads}(\mathrm{CH_3})$; (b) $\Delta E_{ads}(0.5\mathrm{H_2})$ vs. $\Delta E_{ads}(\mathrm{CO})$.

The following equation, defining a linear pyramid-like surface, was used to obtain 2D Sabatier volcano dependencies:

$$\lg\,[TOF(x,y)] = \lg\,(TOF^{opt}) - \max[a_x^+(x-x_0), a_x^-(x_0-x)]$$
$$- \max\left[a_y^+(y-y_0), a_y^-(y_0-y)\right]$$

The tables below list the results of the pyramidal fits. Negative slope values are marked in bold italic typeface. As mentioned above, the only combination of descriptors offering the lowest-RMSE fits and strictly positive $a_{x/y}^{+/-}$ parameters for CH_4 and C2 products is $\Delta E_{ads}(\mathrm{CO})$ and $\Delta E_{ads}(\mathrm{OH})$.

Table S6. Pyramidal fit parameters for CH_4.

Descriptors	$\Delta E_{ads}(\mathrm{OH})$ | $\Delta E_{ads}(\mathrm{CH})$	$E_{ads}(\mathrm{CO})$|$\Delta E_{ads}(\mathrm{CH})$	$E_{ads}(\mathrm{CO})$ | $\Delta E_{ads}(\mathrm{OH})$
R^2	0.804	0.682*	0.779
RMSE, log10	2.329	2.966*	2.476
log10(TOF^{opt})	−8.881	0.703	0.489
x_0	−2.767	−1.089	−1.902
y_0	−4.784	−6.751	−2.767
a_x^+	11.250	15.150	8.130
a_x^-	4.199	7.169	20.209
a_y^+	2.147	4.931	7.644
a_y^-	***−9.355***	2.807	10.344

* The CO-OH combination of descriptors was preferred because of its higher accuracy.

Table S7. Pyramidal fit parameters for CH_3COOH.

Descriptors	ΔE_{ads}(OH) | ΔE_{ads}(CH)	E_{ads}(CO)|ΔE_{ads}(CH)	E_{ads}(CO) | ΔE_{ads}(OH)
R^2	0.826	0.710	0.879
RMSE, log10	1.747	2.257	1.455
log10(TOF^{opt})	−10.807	−8.287	−0.616
x_0	−2.835	−1.062	−2.010
y_0	−4.091	−4.726	−2.767
a_x^+	9.238	15.444	7.082
a_x^-	9.693	1.398	33.065
a_y^+	9.779	0.111	6.761
a_y^-	***−1.815***	***−0.924***	14.079

Table S8. Pyramidal fit parameters for CH_3CHO.

Descriptors	ΔE_{ads}(OH) | ΔE_{ads}(CH)	E_{ads}(CO) | ΔE_{ads}(CH)	E_{ads}(CO) | ΔE_{ads}(OH)
R^2	0.871	0.815*	0.901
RMSE, log10	1.654	1.979*	1.448
log10(TOF^{opt})	−10.443	3.583	1.589
x_0	−2.835	−1.148	−1.984
y_0	−3.868	−6.751	−2.767
a_x^+	9.770	15.726	8.954
a_x^-	1.335	11.488	40.498
a_y^+	1.479	5.835	6.582
a_y^-	***−7.800***	3.823	10.574

* The CO-OH combination of descriptors was preferred because of its higher accuracy.

Table S9. Pyramidal fit parameters for CH_3CH_2OH.

Descriptors	$\Delta E_{ads}(\mathrm{OH})$ | $\Delta E_{ads}(\mathrm{CH})$	$E_{ads}(\mathrm{CO})$|$\Delta E_{ads}(\mathrm{CH})$	$E_{ads}(\mathrm{CO})$ | $\Delta E_{ads}(\mathrm{OH})$
R^2	0.879	0.802*	0.897
RMSE, log10	1.632	2.088*	1.503
log10(TOF^{opt})	−7.919	3.788	0.631
x_0	−2.818	−1.164	−2.020
y_0	−5.474	−6.751	−2.767
a_x^+	10.334	15.709	7.701
a_x^-	1.652	11.651	42.157
a_y^+	1.474	5.806	7.345
a_y^-	***−1.474***	4.814	9.139

* The CO-OH combination of descriptors was preferred because of its higher accuracy.

S6. Additional Discussion of Sabatier Volcano Plots

CO_2 hydrogenation into C_2 products can also produce CH_3CHO. However, we may expect it to be a minor product because of its well-known propensity to undergo reduction into ethanol. The Sabatier volcano for CH_3CHO production on model nanoparticle catalysts is presented in Figure S7.

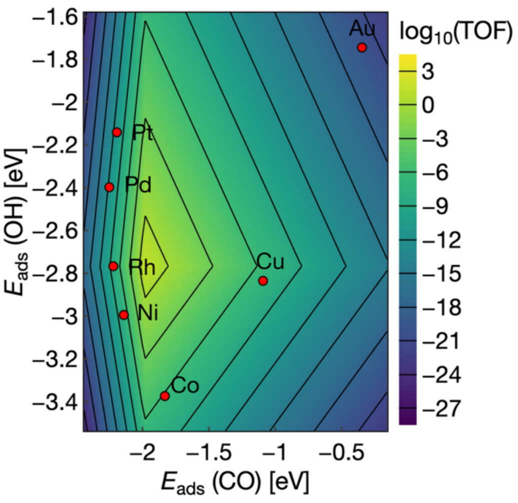

Figure S7. Sabatier volcano plot for CH_3CHO production.

Table S10 presents the parameters of the pyramidal fit for CO_2 hydrogenation into CH_3CHO. The Sabatier-optimal descriptor values $\Delta E_{ads}(\mathrm{CO})$ and $\Delta E_{ads}(\mathrm{OH})$ are similar to those obtained for CH_3COOH and CH_3CH_2OH. This similarity arises because most TOF-determining steps in the pathways leading to CH_3CHO, CH_3COOH, and CH_3CH_2OH involve similar TOF-determining states (see below). Although the Sabatier-optimal TOF^{opt} = 30.88 s^{-1} for CH_3CHO is higher than 4.28 s^{-1} for CH_3CH_2OH, the calculated difference in the Sabatier-optimal catalyst activities is less significant than the RMSD of the fit, which amounts to 28 times. In addition, CH_3CHO can be easily hydrogenated to CH_3CH_2OH (which is the subject of numerous patented catalytic processes), so it is usually not observed as a product of CO_2 hydrogenation. Thus, CH_3CHO may

not be the final product, but rather an intermediate in ethanol synthesis, which would increase the TOF of the latter process.

Table S10. Pyramidal fit parameters for ethanal production.

Product	CH_3CHO
$\Delta E_{ads}(\mathrm{CO})$, eV	−1.98
$\Delta E_{ads}(\mathrm{OH})$, eV	−2.77
TOF^{opt}, s^{-1}	30.88
RMSE $\log_{10}(TOF^{opt})$	1.45

Considering CO, HCOOH, HCHO, and CH_3OH species, each of these (by)products has positive ΔG_r of formation, which is in line with the endergonic formation of CO, HCOOH, HCHO, and CH_3OH from CO_2 and H_2 under many conditions.[23–25] Therefore, the proposed ansatz for the estimation of catalytic activity yields negative TOF (according to equation 1) for the formation of these species under chosen reaction conditions, which would instead facilitate their conversion. As a result, the construction of Sabatier volcanos for CO_2 hydrogenation into these C1 species is not discussed in this study. It should be noted, however, that studies featuring Sabatier volcanoes for the production of methanol via CO_2 hydrogenation are available elsewhere.[26,27]

S7. Representative Mechanisms and Estimated Activities

The results of the calculations within the energetic span model are listed below in Table S11. In this table, the effective TOF of the optimal mechanism is listed along with the TOF-determining intermediate (TDI) and the TOF-determining transition state (TDTS). TDI and TDTS were determined using the degree of TOF control, following the original formalism.[20] Gas-phase methanediol (intermediate 10) was disregarded in the energetic span calculations because of the well-known instability. Below, we give the analysis of the reactivity of Rh nanoparticles that exhibited the highest activity in the formation of ethanol, acetic acid, and methane, followed by the representative mechanisms on other nanoparticles. The full list of automatically found mechanisms is given in the supplementary archive.

Table S11. TOF values of the representative catalytic mechanisms (in s^{-1}), as well as TDI, and elementary steps representing TDTS. TDI and TDTS were determined via the degree of TOF control.[20]

**Metal**		**CH_3CH_2OH**	**CH_3COOH**	**CH_4**
Au	TOF (s^{-1})	$4.92 \cdot 10^{-20}$	$4.74 \cdot 10^{-20}$	$4.93 \cdot 10^{-20}$
	TDI	*[a]	*	*
	TDTS	$CH_2OH^* \longrightarrow CH_2^* + OH^*$	$CH_2OH^* \longrightarrow CH_2^* + OH^*$	$CH_2OH^* \longrightarrow CH_2^* + OH^*$
Co	TOF (s^{-1})	$1.04 \cdot 10^{-6}$	$4.55 \cdot 10^{-11}$	$1.02 \cdot 10^{-6}$
	TDI	HCOO*	CH_3COO^*	HCOO*
	TDTS	$HCOO^* + H^* \longrightarrow HCOOH^*$	$HCOO^* + H^* \longrightarrow HCOOH^*$	$HCOO^* + H^* \longrightarrow HCOOH^*$
Cu	TOF (s^{-1})	$9.35 \cdot 10^{-9}$	$8.02 \cdot 10^{-9}$	$9.47 \cdot 10^{-9}$
	TDI	*	*	*
	TDTS	$CH_2OH^* \longrightarrow CH_2^* + OH^*$	$CH_2OH^* \longrightarrow CH_2^* + OH^*$	$CH_2OH^* \longrightarrow CH_2^* + OH^*$
Ni	TOF (s^{-1})	$2.29 \cdot 10^{-8}$	$4.45 \cdot 10^{-9}$	$4.13 \cdot 10^{-8}$
	TDI	*	CH_2^*	*
	TDTS	$HCOOH^* + H^* \longrightarrow OCH_2OH^*$	$HCOOH^* + H^* \longrightarrow OCH_2OH^*$	$HCOOH^* + H^* \longrightarrow OCH_2OH^*$
Pd	TOF (s^{-1})	$8.77 \cdot 10^{-15}$	$8.39 \cdot 10^{-15}$	$8.87 \cdot 10^{-15}$
	TDI	CO*	CO*	CO*
	TDTS	$CH_2OH^* \longrightarrow CH_2^* + OH^*$	$CH_2OH^* \longrightarrow CH_2^* + OH^*$	$CH_2OH^* \longrightarrow CH_2^* + OH^*$
Pt	TOF (s^{-1})	$1.97 \cdot 10^{-11}$	$7.35 \cdot 10^{-10}$	$1.89 \cdot 10^{-9}$
	TDI	CO*	CO*	CO*
	TDTS	$CH_3CH_2O^* + H^* \longrightarrow CH_3CH_2OH^*$	$CH_3COO^* + H^* \longrightarrow CH_3COOH^*$	$CH_2O^* + H^* \longrightarrow CH_2OH^*$
Rh	TOF (s^{-1})	$1.03 \cdot 10^{-5}$	$1.0 \cdot 10^{-6}$	$1.24 \cdot 10^{-2}$
	TDI	CH*	CH*	*
	TDTS	$HCOO^* + H^* \longrightarrow HCOOH^*$	$HCOO^* + H^* \longrightarrow HCOOH^*$	$HCOO^* + H^* \longrightarrow HCOOH^*$

[a] Unoccupied active site is the TDI.

Selected mechanisms on Rh nanoparticles. On Rh nanoparticles, the mechanisms of formation of all four target products, CH_3COOH, CH_3CHO, CH_3CH_2OH, and CH_4, share a common sequence of initial elementary steps transforming CO_2 to CH_x. The mechanisms begin with CO_2 adsorption and hydrogenation along the formate pathway:

$$CO_2 \rightarrow CO_2* \rightarrow HCOO* \rightarrow HCOOH* \rightarrow CHO* \rightarrow CHOH* \rightarrow CH* \rightarrow CH_2*.$$

The preference for the formate pathway is clear when we compare the activation barriers in the formation of HCOO* ($\Delta G^{\ddagger}$ = 0.66 eV) *vs.* the formation of a carboxyl-like HOCO* from CO_2* ($\Delta G^{\ddagger}$ = 0.91 eV). Once HCOO* is formed, its protonation to HCOOH* exhibits a high barrier ($\Delta G^{\ddagger}$ = 1.06 eV), whereas the subsequent steps along the common pathway are kinetically more facile. Accordingly, our automated analysis of the reaction graph consistently identifies the protonation:

$$HCOO* + H* \rightarrow HCOOH*$$

as the TDTS for all four products.

The subsequent transformation converts formic acid into strongly-binding CH*. In the reaction network on Rh, CH* is one of the most stable surface intermediates, with a relative free energy of approximately −0.57 eV. This deep minimum makes CH* the resting state of the catalytic cycles that lead to C2 oxygenates in the modeled network. Accordingly, within the energetic span model applied to the optimal pathways, CH* is the TOF-determining intermediate for CH_3COOH*, CH_3CHO*, and CH_3CH_2OH*. We can expect high surface concentration of these strongly bound species, whereas the dominant kinetic bottleneck is the earlier protonation of formate. Although some late steps, such as the protonation of CH_3COO* to CH_3COOH*, also involve relatively high barriers (in this exemplary case, $\Delta G^{\ddagger}$ = 1.24 eV), they lie closer to the product side of an overall exergonic cycle and do not depress the TOF value.

For CH_3COOH^*, the optimal continuation of the CH_2^* transformation under H_2/CO_2 feed is:

$$CH_2^* \rightarrow CH_2COO^* \rightarrow CH_3COO^* \rightarrow CH_3COOH^*,$$

With an overall reaction free energy $\Delta G_r = -0.15$ eV and an estimated TOF of approximately 1×10^{-6} s^{-1}. After the common CO_2 to CH_2^* sequence, C–C coupling of CH_2^* with CO_2^* forms CH_2COO^* with a barrier of about 0.82 eV, which is higher than the further hydrogenation of CH_2^* to CH_4 (see below). Subsequent hydrogenation of CH_2COO^* to CH_3COO^* is relatively facile ($\Delta G^\ddagger = 0.31$ eV), whereas the final $CH_3COO^* \rightarrow CH_3COOH^*$ step is kinetically hampered ($\Delta G^\ddagger = 1.24$ eV). Nevertheless, because this late barrier is situated close to the product and the overall cycle is moderately exergonic, the energetic span analysis assigns a smaller degree of TOF control to this step than to $HCOO^* \rightarrow HCOOH^*$.

The CH_3CHO^* and $CH_3CH_2OH^*$ formation mechanisms under H_2/CO_2 feed extend the acetic acid pathway. For CH_3CHO^*, the optimal pathway continues as:

$$CH_3COOH^* \rightarrow CH_3CH(O)OH^* \rightarrow CH_3CHO^*,$$

With overall ($CO_2 \rightarrow CH_3CHO$) $\Delta G_r = -0.21$ eV and TOF = 3.9×10^{-6} s^{-1}. Starting from CH_3COOH^*, the sequence proceeds with the hydrogenation into $CH_3CH(O)OH^*$, followed by C–O cleavage to give surface-bound CH_3CHO^*. The formation of $CH_3CH(O)OH^*$ is strongly uphill in free energy ($\Delta G = +1.0$ eV) and passes through a barrier of about 1.05 eV, indicating that further reduction of acetic acid is kinetically unfavorable on Rh. However, the subsequent C–O cleavage is nearly barrierless ($\Delta G^\ddagger = 0.03$ eV) and exergonic ($\Delta G = -0.15$ eV). For $CH_3CH_2OH^*$, the mechanism continues from CH_3CHO^* as:

$$CH_3CHO^* \rightarrow CH_3CH_2O^* \rightarrow CH_3CH_2OH^*,$$

With overall ($CO_2 \rightarrow CH_3CH_2OH$) $\Delta G_r = -0.26$ eV and TOF = 1.0×10^{-5} s^{-1}. Hydrogenation of CH_3CHO^* to the ethoxide intermediate $CH_3CH_2O^*$ and its subsequent protonation to

CH_3CH_2OH* proceed over moderate barriers equal to 0.76 and 0.75 eV, respectively. Thus, once CH_3CHO* is formed, the additional steps to ethanol are more kinetically feasible than the upstream steps of HCOO* protonation and endergonic transformation of CH_3COOH* to $CH_3CH(O)OH$*. The analysis within the energetic span model thus rationalizes why the optimal TOFs for CH_3CHO* and CH_3CH_2OH* are of similar magnitude and why CH* is the TDI for the C2 oxygenates under discussion.

Methane formation follows a different branch after CH_2*:

$$CH_2^* \rightarrow CH_3^* \rightarrow CH_4^*,$$

With the overall ΔG_r of -1.12 eV, indicating a highly thermodynamically favorable process, and a significantly higher TOF of 1.2×10^{-2} s^{-1}. From CH_2*, the lowest-barrier pathway in the network is hydrogenation to CH_3* (0.69 eV), which is followed by the facile hydrogenation to CH_4* (0.59 eV). Both steps have lower barriers than the C-C coupling steps that initiate the C2 branches. Given the strongly exergonic overall formation of methane, the energetic span model shifts the TDI from the deep CH* well to the initial state, CO_2 over the catalyst surface, while retaining formate protonation as the TDTS. Accordingly, Rh is predicted to be significantly more active in methane production than in the synthesis of C2 oxygenates, even though the local barriers beyond CH_2* are moderate in both branches.

We evaluated the kinetic graphs under an alternative scenario with finite initial costs for all gas-phase species (HCOOH, CO, CH_2O, CH_3OH, CH_4, CH_3COOH, CH_3CHO, and CH_3CH_2OH, each at a pressure of 1 bar) to model the catalytic process under operating conditions in which notable amounts of gas products have already accumulated. Such a scenario does not change the TDI-TDTS pairs but alters the optimal mechanisms for CH_3CHO and CH_3CH_2OH. For acetic acid and methane, the mechanisms remain identical to the case where CO_2 was the only carbon source in

the reactant feed, reflecting the fact that both still start from CO_2 and proceed along the formate pathway. In contrast, for CH_3CHO and CH_3CH_2OH, the lowest-cost cycles now avoid the strongly uphill CH_3COOH^* → $CH_3CH(O)OH^*$ step. The algorithm instead identified routes in which CH_2^* couples directly to available CH_2O:

$$CH_2^* \rightarrow \text{-}CH_2CH_2O\text{-}^* \rightarrow CH_3CH_2O^* \rightarrow CH_3CHO^*$$

$$CH_2^* \rightarrow \text{-}CH_2CH_2O\text{-}^* \rightarrow CH_3CH_2O^* \rightarrow CH_3CH_2OH^*.$$

The key step of the C–C coupling between CH_2^* and CH_2O^* proceeds via a moderate barrier of 1.02 eV. Because CH_2O^* species are now "cheap" in terms of compound costs, these cycles become favored, although some individual barriers are slightly higher than along the purely CO_2-driven route. Accounting for the supply of CH_2O^* reorganizes the reaction mechanism rather locally (in particular, it facilitates direct C–C bond formation from CH_2^* and CH_2O^*); however, it does not change the key kinetic bottleneck, the formation of CH_2^* species from CO_2.

On **Au nanoparticles**, the optimal mechanisms for CH_3COOH, CH_3CHO, CH_3CH_2OH, and CH_4 follow the same initial sequence of elementary steps:

$$CO_2 \rightarrow CO_2^* \rightarrow HCOO^* \rightarrow HCOOH^* \rightarrow OCH_2OH^* \rightarrow CH_2O^* \rightarrow CH_2OH^* \rightarrow CH_2^*,$$

That is, the formate pathway followed by reduction through a methanediolate-formaldehyde-methoxy sequence. As on Rh, the initial hydrogenation of CO_2^* to $HCOO^*$ is clearly preferred over the branch involving carboxyl-like $HOCO^*$ (0.79 vs. 1.41 eV). The analysis within the energetic span identified the C–O cleavage in CH_2OH^* yielding CH_2^* as the TDTS for all four products, with a barrier of 2.08 eV and a strongly endergonic character (+1.54 eV). All surface intermediates along the pathway, including $HCOO^*$, $HCOOH^*$, CH_2O^*, and CH_2^*, remain above the reference free energy, so the initial CO_2 over the clean surface acts as the TDI. As a result, the effective TOF is driven by the free energy difference between the TS energy in the C–O cleavage

in CH_2OH^* and the initial state, and all products inherit extremely low TOFs of order 10^{-20} s^{-1}. Further branching elementary steps after CH_2^* to either CH_3COOH, CH_3CHO, CH_3CH_2OH, or simply CH_4 involve moderate barriers ($\Delta G^{\ddagger}$ = 0.3-1.1 eV) and therefore do not alter the TDI/TDTS pair. In the scenario in which all gas-phase species have finite initial costs, the mechanism for CH_3COOH and CH_4 is unchanged, whereas CH_3CHO and CH_3CH_2OH formation is rerouted through coupling between CH_2^* and CH_2O^*.

On **fcc-Co nanoparticles**, the CO_2 hydrogenation mechanisms show a stronger resemblance to those on Rh, but with important differences in the resting states and kinetically preferred C–C coupling steps. The lowest-cost paths for all products begin with CO_2 activation via the formate pathway:

$CO_2 \rightarrow CO_2^* \rightarrow HCOO^* \rightarrow HCOOH^* \rightarrow CHO^* \rightarrow CHOH^* \rightarrow CH_2OH^* \rightarrow CH_2^*$,

With CO_2^* hydrogenation into $HCOO^*$ having the free energy of activation equal to 0.68 eV. The competing route involving CO_2 transformation into $HOCO^*$ has a very high barrier (1.74 eV), so it is kinetically disfavored. Within the optimal mechanism, the protonation of $HCOO^*$ to $HCOOH^*$ remains the dominant kinetic bottleneck and serves as TDTS for all products with $\Delta G^{\ddagger}$ = equal to 1.72 eV, which is significantly higher than those of downstream elementary steps. The TDI depends on the target product, while being either the surface-adsorbed formate or acetate, which reflects the relatively high electropositivity of Co.

For CH_3COOH, the reaction pathways continue via:

$$CH_2^* \rightarrow CH_2COO^* \rightarrow CH_3COO^* \rightarrow CH_3COOH^*.$$

The acetate CH_3COO^* is relatively stable on fcc-Co (relative ΔG = −0.88 eV) and emerges as the TDI. For CH_3CHO and CH_3CH_2OH, the optimal paths do not include CH_2-CO_2 coupling and

proceed via CH_2-CO coupling instead, which changes the TDI to HCOO* (relative $\Delta G = -0.28$ eV).

On **Cu nanoparticles**, the mechanisms also follow the formate-based pathway, with the initial sequence being:

$$CO_2 \rightarrow CO_2^* \rightarrow HCOO^* \rightarrow HCOOH^* \rightarrow OCH_2OH^* \rightarrow CH_2O^* \rightarrow CH_2OH^* \rightarrow CH_2^*.$$

In line with Cu being a weakly-binding metal, the TDI is the initial state (gaseous CO_2). The analysis of the mechanisms on Cu assigned TDTS to $CH_2OH^* \rightarrow CH_2^*$ ($\Delta G^{\ddagger} = 0.85$ eV). The relatively high activation energy of this step will facilitate CO_2 hydrogenation to methanol on Cu catalysts (at appropriately chosen reaction conditions). Most intermediates in the network are well above the reference free energy, and even the strongly binding species, such as CH_3COO^*, have only weakly exergonic formation energies ($\Delta G = -0.05$ eV).

Branching after CH_2^* on Cu is similar to the mechanistic picture on Rh nanoparticles, but with different preferred channels. Under CO_2 hydrogenation feed, CH_3COOH is formed via:

$$CH_2^* \rightarrow CH_2COO^* \rightarrow CH_3COO^* \rightarrow CH_3COOH^*,$$

while CH_3CHO and CH_3CH_2OH are accessed by further reduction of CH_3COOH^*. When all gas-phase species are considered to be present in the reaction mixture and have finite starting costs, acetic acid and methane cycles remain essentially unchanged, whereas the CH_3CHO and CH_3CH_2OH mechanisms reroute through $CH_2^* + CH_2O^*$ coupling. Finally, it should be noted that the automatically found pathways mirror our previous detailed mechanistic analysis of CO_2 hydrogenation pathways on Cu nanoparticles based on manual comparison of free activation energies.[8]

On **Ni nanoparticles**, the optimal mechanisms again share the formate-involving sequence of initial elementary steps:

$CO_2 \rightarrow CO_2* \rightarrow HCOO* \rightarrow HCOOH* \rightarrow OCH_2OH* \rightarrow CH_2O* \rightarrow CH_2OH* \rightarrow CH_2*$.

CO_2* hydrogenation into HCOO* is preferred over HOCO*, because of a lower activation barrier ($\Delta G^{\ddagger}$ = 0.88 *vs.* 1.0 eV). The analysis of the degree of TOF control within the energetic span model identified HCOOH* → OCH_2OH* as the TDTS for all four products, with $\Delta G^{\ddagger}$ = 0.65 eV and a substantially positive reaction free energy (+0.22 eV). Relatively stable CH_2 (relative ΔG = −0.24 eV) serves as TDI for the formation of CH_3COOH and CH_3CHO on Ni, whereas the bare catalyst surface is TDI for CH_3CH_2OH and CH_4.The reaction pathways after the formation of CH_2* are analogous to many previous cases. Under CO_2 hydrogenation feed, CH_3COOH forms via CH_2*-CO_2* coupling, while CH_3CHO and CH_3CH_2OH are formed by further reduction of acetic acid. In the scenario when all gas-phase species have finite initial costs, the acetic acid and methane production cycles remain essentially unchanged, while the mechanisms for CH_3CHO and CH_3CH_2OH formation involve CH_2*-CH_2O* coupling that yields -CH_2CH_2O-*. The latter is subsequently converted to CH_3CH_2O* and onward to CH_3CHO* (dehydrogenation) or CH_3CH_2OH (protonation).

On **Pd and Pt nanoparticles**, the activation barriers of the early hydrogenation steps and the relative free energies of C1 intermediates lead to a qualitatively different mechanistic picture. In contrast to Rh, Co, Cu, Ni, and Au, the hydrogenation of CO_2* to HCOO* is less favorable than the formation of carboxyl-like HOCO* ($\Delta G^{\ddagger}$ = 0.96 *vs.* 0.78 eV on Pd; $\Delta G^{\ddagger}$ = 1.12 *vs.* 0.73 eV on Pt), and the subsequent HCOOH* intermediate is both less stable than CO* and significantly more expensive in terms of PATHFINDER compound costs (see full data in the supplementary ZIP archive). At the same time, CO* is most stable among the C1 intermediates in the optimal pathways found on Pd and Pt nanoparticles, so under stationary conditions the surface is expected to be dominated by CO* rather than by formate. If the reaction network includes the formate

branch in the graph-based analysis, the algorithm finds formally low-cost paths producing CH_2* via HCOO* and HCOOH* intermediates. However, these pathways implicitly assume non-negligible coverages of high-energy formate species that are thermodynamically and kinetically disfavored relative to CO*. To enforce a mechanistic picture that is consistent with both lower barriers for CO formation, the compound-cost ranking, and the known propensity of Pd and Pt to accumulate CO*, we rerun the analysis with suppressed CO_2* → HCOO* and HCOO* → HCOOH* transitions in the network. This procedure reorients the optimal cycles to pass through the CO_2* → HOCO* → CO* branch and ensures that the energetic span is evaluated relative to a CO*-rich surface state rather than the transient formate pool. In other words, the modified network can be viewed as a coarse-grained representation where the rare formate channel is excluded in favor of a more realistic CO_2 ⇄ CO quasi-equilibrium, in line with well-known facile CO* formation on Pd and Pt.

For **Pd** nanoparticles, the algorithm identified the following sequence of initial steps:

$$CO_2 \rightarrow CO_2^* \rightarrow HOCO^* \rightarrow CO^* \rightarrow CHO^* \rightarrow CH_2O^* \rightarrow CH_2OH^* \rightarrow CH_2^*,$$

Which branches at CH_2* via CH_2COO* → CH_3COO* → CH_3COOH* to acetic acid. The latter can be further hydrogenated to CH_3CHO* (i.e., *via* CH_3COOH* → $CH_3CH(O)OH$* → CH_3CHO*), and CH_3CH_2OH* is formed through the hydrogenation of CH_3CHO* via CH_3CH_2O* intermediate. In turn, methane formation proceeded *via* the subsequent hydrogenation of CH_2* to CH_3* and CH_4*. CO* was the TDI for all products, while the TS in CH_2OH* → CH_2* remained the TDTS in all identified optimal cycles. The computed TOF values were as low as 10^{-14} and 10^{-15} s^{-1}, which is strongly affected by the energy required to remove highly stable CO* species poisoning the surface of Pd. Thus, any mechanism that bypasses CO* as the resting state of Pd would grossly overestimate its activity in CO_2 hydrogenation. Indeed, the automatically assigned

pathway through formate intermediate with CH_2OH^* → CH_2^* as the TDTS and bare catalyst surface as TDI has a TOF of 10^{-6} s^{-1} due to the neglected effect of poisoning by off-cycle CO intermediates. In that sense, the automated analysis of pristine catalytic network overestimated TOFs on Pd by neglecting the CO coverage penalty, whereas the modified network restored the kinetic impact of CO poisoning while preserving the C–O cleavage in CH_2OH^* as the key chemical bottleneck.

On **Pt nanoparticles**, when all elementary steps are retained, the automated analysis identified formate-based pathways for all C2 products and methane, starting with the following sequence of steps:

$$CO_2 \rightarrow CO_2^* \rightarrow HCOO^* \rightarrow HCOOH^* \rightarrow CHO^* \rightarrow CH_2O^* \rightarrow CH_2OH^* \rightarrow CH_2^*,$$

In this case, the analysis within the energetic span model assigned $HCOO^*$ → $HCOOH^*$ as the TDTS-bearing step for CH_3COOH^*, CH_3CHO^*, and $CH_3CH_2OH^*$, with CH_2^* as the TDI, while for CH_4, the initial state is the TDI, and the TDTS remains the same. The resulting TOFs are modest (from about 10^{-11} to 10^{-10} s^{-1} for C2 products and 10^{-8} s^{-1} for CH_4). Disallowing the CO_2^* → $HCOO^*$ and $HCOO^*$ → $HCOOH^*$ transitions reoriented the Pt network toward the more plausible CO_2^* → $HOCO^*$ → CO^* branch. In the modified analysis, the optimal cycles for all products begin with

$$CO_2 \rightarrow CO_2^* \rightarrow HOCO^* \rightarrow CO^* \rightarrow CHO^* \rightarrow CH_2O^* \rightarrow CH_2OH^* \rightarrow CH_2^*,$$

And CO^* becomes the TDI, in agreement with its role as the most stable C1 intermediate. The TDTS, however, is now product-specific and situated later in the cycle: for CH_3COOH^* it is the TS in CH_3COO^* → CH_3COOH^*, for CH_3CHO^* it is in CH_3COOH^* → $CH_3CH(O)OH^*$, for $CH_3CH_2OH^*$ it is in $CH_3CH_2O^*$ → $CH_3CH_2OH^*$, and for CH_4^* it is in CH_2O^* → CH_2OH^*. The

corresponding TOF values changed as well: methane formation obtained a reduced TOF of 10^{-9} s^{-1}, whereas the C2 products exhibited TOFs in the range of 10^{-11} to 10^{-10} s^{-1}.

In summary, the found optimal pathways on Pd and Pt nanoparticles highlight the importance of aligning graph-based mechanistic analysis with realistic surface coverage. For both metals, the automated pathway search algorithm is biased toward formate-based cycles that do not account for the strong thermodynamic and kinetic preference for CO* formation and accumulation. Once the formate branch is suppressed to reflect the fact that CO* is both kinetically preferred and thermodynamically "cheaper" than HCOOH*, the resulting mechanisms pass through CO* as the TDI and yield substantially lower TOFs on Pd and Pt.

S8. Microkinetic Simulations

Microkinetic simulations were conducted using the MKMCXX program[28] at similar reaction conditions, T = 523.15 K, p(H_2) = 30 bar, p(CO_2) = 10 bar. Since the primary effect of temperature is on the rates of adsorption and desorption rather than on surface reactions, we sampled the process at additional temperatures of 450, 475, 500, and 550 K to better understand the system's behavior under conditions typical of CO_2 hydrogenation. The corresponding production rates, however, contain a bias due to the use of constant activation barriers and fixed Eyring pre-exponential factors calculated for 523.15 K, which decreases the accuracy of microkinetic simulations at temperatures below 500 K. Only the values calculated at T = 523.15 K are discussed in the main text. The forward and reverse free activation energies presented in the supporting spreadsheet were used in the simulations. The Eyring preexponential of $1.0901 \cdot 10^{13}$ s^{-1} (T = 523.15 K) was used for all surface reactions. All systems were simulated for 864000.0 s (10 days). Adsorbate surface areas (A) were calculated assuming the largest possible cross-section of the van der Waals volumes formed using Bondi radii. Parameters used in the calculation of adsorption/desorption rates (surface area of the adsorbed specie, A; molecular weight, M_r; rotational temperature, θ_{rot}; rotational symmetry number, σ) are presented in Table S12 below. All elementary steps in the microkinetic models and the corresponding energetic parameters are listed in the Supplementary Spreadsheet.

Figure S8a shows the production rates on the Rh nanoparticle. Evidently, the only product formed is HCOOH, which is readily desorbed. Figure S8b illustrates a scenario in which the desorption of most C1 products and byproducts leading to ethanol and acetic acid was blocked by removing the corresponding adsorption/desorption equilibria. This modification resulted in the formation of ethanol exclusively, gradually increasing and reaching $1.12 \cdot 10^{-5}$ s^{-1} at T = 550 K.

Table S12. Parameters used in the calculations of adsorption/desorption rates.

Adsorbate	A [m^2]	M_r	θ_{rot}	σ
CO_2	9.819E-20	44.009	0.541	2
HCOOH	1.340E-19	46.025	0.487	1
CH_2O	1.034E-19	30.026	1.604	2
$HOCH_2OH$	1.563E-19	48.041	0.421	2
CO	8.173E-20	28.01	2.716	1
CH_3OH	1.370E-19	32.042	1.122	1
CH_3COOH	2.244E-19	60.052	0.251	1
CH_3CHO	1.742E-19	44.053	0.429	1
CH_3CH_2OH	1.858E-19	46.069	0.384	1
CH_4	9.913E-20	16.043	7.449	12
H_2	4.675E-20	2.016	85.929	2
H_2O	7.458E-20	18.015	13.396	2

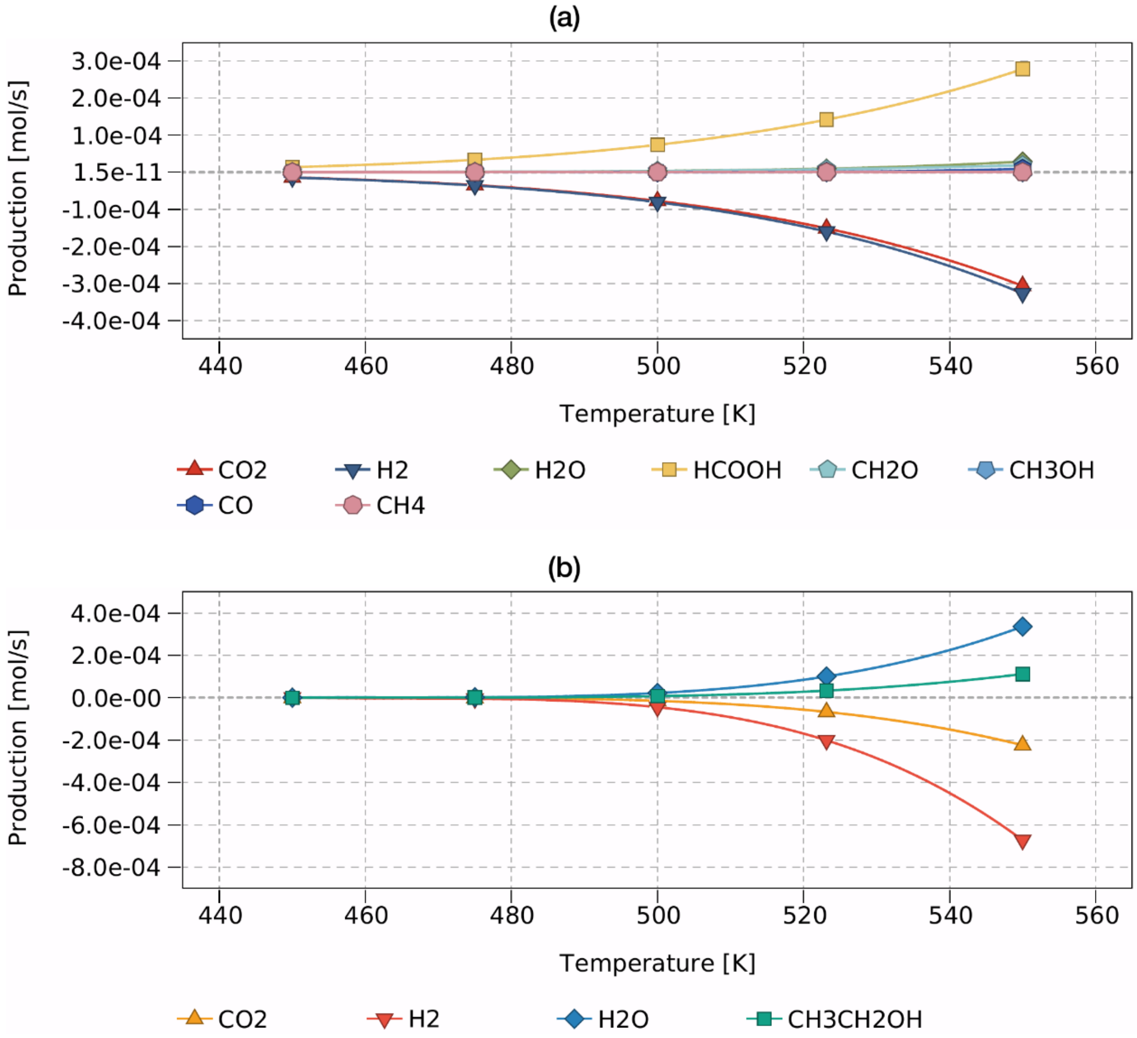

Figure S8. Production rates in microkinetic simulations of CO_2 hydrogenation on: (a) Rh nanoparticle; (b) Rh nanoparticle with adsorption/desorption of most C1 products and byproducts leading to ethanol and acetic blocked.

S9. References

[1] G. Kresse, J. Furthmüller, "Efficient iterative schemes for *ab initio* total-energy calculations using a plane-wave basis set" *Phys. Rev. B* **1996**, *54*, 11169–11186.

[2] Y. Zhang, W. Yang, "Comment on 'Generalized Gradient Approximation Made Simple'" *Phys. Rev. Lett.* **1998**, *80*, 890.

[3] P. Janthon, S. Luo, S. M. Kozlov, F. Viñes, J. Limtrakul, D. G. Truhlar, F. Illas, "Bulk properties of transition metals: A challenge for the design of universal density functionals" *J. Chem. Theory Comput.* **2014**, *10*, 3832–3839.

[4] L. Goerigk, A. Hansen, C. Bauer, S. Ehrlich, A. Najibi, S. Grimme, "A look at the density functional theory zoo with the advanced GMTKN55 database for general main group thermochemistry, kinetics and noncovalent interactions" *Physical Chemistry Chemical Physics* **2017**, *19*, 32184–32215.

[5] G. Kresse, D. Joubert, "From ultrasoft pseudopotentials to the projector augmented-wave method" *Phys. Rev. B* **1999**, *59*, 1758–1775.

[6] S. Grimme, J. Antony, S. Ehrlich, H. Krieg, "A consistent and accurate *ab initio* parametrization of density functional dispersion correction (DFT-D) for the 94 elements H-Pu" *J. Chem. Phys.* **2010**, *132*, 154104.

[7] S. Grimme, S. Ehrlich, L. Goerigk, "Effect of the damping function in dispersion corrected density functional theory" *J. Comput. Chem.* **2011**, *32*, 1456–1465.

[8] M. V Polynski, S. M. Kozlov, "Reaction Network of CO_2 Hydrogenation into C1-2 Oxygenates and Its BEP Relationships" *EES Catalysis* **2026**, DOI 10.1039/D5EY00338E.

[9] E. Bitzek, P. Koskinen, F. Gähler, M. Moseler, P. Gumbsch, "Structural relaxation made simple" *Phys. Rev. Lett.* **2006**, *97*, 170201.

[10] G. Henkelman, H. Jónsson, "A dimer method for finding saddle points on high dimensional potential surfaces using only first derivatives" *J. Chem. Phys.* **1999**, *111*, 7010–7022.

[11] A. Heyden, A. T. Bell, F. J. Keil, "Efficient methods for finding transition states in chemical reactions: Comparison of improved dimer method and partitioned rational function optimization method" *J. Chem. Phys.* **2005**, *123*, 224101.

[12] J. Kästner, P. Sherwood, "Superlinearly converging dimer method for transition state search" *J. Chem. Phys.* **2008**, *128*, 014106.

[13] P. Xiao, D. Sheppard, J. Rogal, G. Henkelman, "Solid-state dimer method for calculating solid-solid phase transitions" *J. Chem. Phys.* **2014**, *140*, 174104.

[14] A. Hjorth Larsen, J. JØrgen Mortensen, J. Blomqvist, I. E. Castelli, R. Christensen, M. Dułak, J. Friis, M. N. Groves, B. Hammer, C. Hargus, E. D. Hermes, P. C. Jennings, P. Bjerre Jensen, J. Kermode, J. R. Kitchin, E. Leonhard Kolsbjerg, J. Kubal, K. Kaasbjerg, S. Lysgaard, J. Bergmann Maronsson, T. Maxson, T. Olsen, L. Pastewka, A. Peterson, C. Rostgaard, J. SchiØtz, O. Schütt, M. Strange, K. S. Thygesen, T. Vegge, L. Vilhelmsen, M. Walter, Z. Zeng, K. W. Jacobsen, "The atomic simulation environment—a Python library for working with atoms" *Journal of Physics: Condensed Matter* **2017**, *29*, 273002.

[15] Y. Sheng, M. V. Polynski, M. K. Eswaran, B. Zhang, A. M. H. Lim, L. Zhang, J. Jiang, W. Liu, S. M. Kozlov, "A review of mechanistic insights into CO_2 reduction to higher alcohols for rational catalyst design" *Appl. Catal. B* **2024**, *343*, 123550.

[16] M. G. Evans, M. Polanyi, "Some applications of the transition state method to the calculation of reaction velocities, especially in solution" *Transactions of the Faraday Society* **1935**, *31*, 875–894.

[17] L. Prokhorenkova, G. Gusev, A. Vorobev, A. V. Dorogush, A. Gulin in *Advances in Neural Information Processing Systems 31 (NeurIPS 2018)*, **2018**.

[18] L. Himanen, M. O. J. Jäger, E. V. Morooka, F. Federici Canova, Y. S. Ranawat, D. Z. Gao, P. Rinke, A. S. Foster, "DScribe: Library of descriptors for machine learning in materials science" *Comput. Phys. Commun.* **2020**, *247*, 106949.

[19] S. Kozuch, "Steady State Kinetics of Any Catalytic Network: Graph Theory, the Energy Span Model, the Analogy between Catalysis and Electrical Circuits, and the Meaning of 'Mechanism'" *ACS Catal.* **2015**, *5*, 5242–5255.

[20] S. Kozuch, "A refinement of everyday thinking: the energetic span model for kinetic assessment of catalytic cycles" *Wiley Interdiscip. Rev. Comput. Mol. Sci.* **2012**, *2*, 795–815.

[21] P. L. Türtscher, M. Reiher, "Pathfinder─Navigating and Analyzing Chemical Reaction Networks with an Efficient Graph-Based Approach" *J. Chem. Inf. Model.* **2022**, *63*, 147–160.

[22] A. A. Hagberg, D. A. Schult, P. J. Swart in *Proceedings of the 7th Python in Science Conference (SciPy 2008)* (Eds.: G. Varoquaux, T. Vaught, J. Millman), SciPy, **2008**, p. 16.

[23] A. Cárdenas-Acero, Cristian Álvarez-Romero, Carlos Daza, A. Álvarez, E. A. Baquero, "Exploring heterogeneous Ru-based catalysts: CO2 hydrogenation towards formic acid, formaldehyde, and methanol" *Discover Catalysis* **2024**, *1*, 1–39.

[24] M. A. Nolen, S. A. Tacey, S. Kwon, C. A. Farberow, "Theoretical assessments of CO2 activation and hydrogenation pathways on transition-metal surfaces" *Appl. Surf. Sci.* **2023**, *637*, 157873.

[25] Z. Wei, X. Tian, M. Bender, M. Beller, H. Jiao, "Mechanisms of CoIIand Acid Jointly Catalyzed Domino Conversion of CO2, H2, and CH3OH to Dialkoxymethane: A DFT Study" *ACS Catal.* **2021**, *11*, 6908–6919.

[26] S. J. Han, J. Chen, H. G. Park, K. W. Jun, S. K. Kim, "Optimizing Fe-based catalysts for CO2 hydrogenation using combined theoretical predictions and experimental insights" *Chemical Engineering Journal* **2025**, *508*, 161006.

[27] S. Polierer, J. Jelic, S. Pitter, F. Studt, "On the Reactivity of the Cu/ZrO2 System for the Hydrogenation of CO2 to Methanol: A Density Functional Theory Study" *Journal of Physical Chemistry C* **2019**, *123*, 26904–26911.

[28] I. A. W. Filot, R. A. Van Santen, E. J. M. Hensen, "The Optimally Performing Fischer–Tropsch Catalyst" *Angew. Chem. Int. Ed.* **2014**, *53*, 12746–12750.
